# Cellular uptake of a cystine-knot peptide and modulation of its intracellular trafficking

**DOI:** 10.1038/srep35179

**Published:** 2016-10-13

**Authors:** Xinxin Gao, Karen Stanger, Harini Kaluarachchi, Till Maurer, Paulina Ciepla, Cecile Chalouni, Yvonne Franke, Rami N. Hannoush

**Affiliations:** 1 Department of Early Discovery Biochemistry, Genentech, South San Francisco, California; 2 Department of Structural Biology, Genentech, South San Francisco, California; 3 Department of Pathology, Genentech, South San Francisco, California

## Abstract

Cyclotides or cyclic cystine-knot peptides have emerged as a promising class of pharmacological ligands that modulate protein function. Interestingly, very few cyclotides have been shown to enter into cells. Yet, it remains unknown whether backbone cyclization is required for their cellular internalization. In this report, we studied the cellular behavior of EETI-II, a model acyclic cystine-knot peptide. Even though synthetic methods have been used to generate EETI-II, recombinant methods that allow efficient large scale biosynthesis of EETI-II have been lagging. Here, we describe a novel protocol for recombinant generation of folded EETI-II in high yields and to near homogeneity. We also uncover that EETI-II is efficiently uptaken via an active endocytic pathway to early endosomes in mammalian cells, eventually accumulating in late endosomes and lysosomes. Notably, co-incubation with a cell-penetrating peptide enhanced the cellular uptake and altered the trafficking of EETI-II, leading to its evasion of lysosomes. Our results demonstrate the feasibility of modulating the subcellular distribution and intracellular targeting of cystine-knot peptides, and hence enable future exploration of their utility in drug discovery and delivery.

The cystine-knot family of peptides has generated interest as a promising scaffold for drug design and discovery. These peptides contain approximately 30 amino acid residues, comprising conserved cysteines that form intramolecular disulfide bonds arranged in a knotted conformation known as the cystine-knot motif^1^. The arrangement of the cystine-knot, along with the identity of the amino acid sequence, confers exceptional stability to the framework against thermal, chemical and enzymatic degradation^2^. Moreover, the high diversity present in the loop regions flanking the conserved cysteine residues implies that non-native sequences might be tolerated in these regions without affecting the overall knotted fold. Indeed, multiple cystine-knot backbones have been used as frameworks for peptide grafting, successfully incorporating novel biological function through the grafted peptide while maintaining the stable cystine-knot structure^3^,^4^,^5^,^6^. Together these properties hold promise for the use of cystine-knot peptides in drug discovery.

A class of cystine-knot peptides, referred to as cyclic cystine-knot (CCK) peptides or cyclotides^7^, is exclusively found in plants and features a head-to-tail cyclized backbone, resulting in peptides that lack free N- or C-termini. Cyclotides typically fall into one of two subfamilies, Möbius and bracelet, depending on the presence or absence of a twist in the cyclic backbone caused by a cis-Pro amide bond, respectively^7^. Additionally, a third cyclotide subfamily contains just two members, MCoTI-I and MCoTI-II, which were isolated from the seeds of the fruit *Momordica Cochinchinensis,* a member of the Cucurbitaceae plant family^8^. The MCoTI cyclotides are potent trypsin inhibitors and despite their cyclic backbone, are closer in sequence and structure to the EETI family of acyclic cystine-knot peptides, known as the squash trypsin inhibitors, than they are to prototypic Möbius or bracelet cyclotides^9^. In general, the role of the cyclic backbone of cyclotides has remained controversial and appears to be context-dependent. For example, the cyclic backbone in kalata B1, a prototypical cyclotide (Fig. 1a), seems to play a role in mediating its anti-HIV activity and its physical interactions with components of the phospholipid bilayer^2^,^10^,^11^,^12^. On the other hand, recent studies demonstrated that backbone cyclization does not seem to contribute strongly to the chemical stability of MCoTI-II cyclotides and their trypsin inhibitory bioactivity^13^,^14^.

Interestingly, it was shown that the cyclotides MCoTI-I, MCoTI-II and kalata B1 are internalized into cells via overlapping yet distinct mechanisms^15^,^16^,^17^. The MCoTI family is internalized into mammalian cells primarily through macropinocytosis (fluid phase uptake)^16^,^17^, an endocytic process that is typically dependent on the actin cytoskeleton and results in the formation of large endocytic vesicles known as macropinosomes. Although to a lesser extent, MCoTI-I uptake also involves other endocytic pathways such as cholesterol-dependent and clathrin-mediated endocytosis^17^, with all internalization routes eventually leading to the late endosome and lysosomal compartments. While the internalization route of kalata B1 (prototypic of the Möbius family) is not entirely clear, this cyclotide binds to the phosphatidylethanolamine phospholipid component of membranes, likely leading to cellular penetration through direct membrane interaction^16^,^18^,^19^. The differences in cellular internalization likely stem from the different propensities of each cyclotide to recognize and bind various components of the phospholipid bilayer^16^,^20^. Clearly, further work is needed to develop a more comprehensive understanding of the nature of the interaction of the different cyclotides with the cellular lipid membrane and the intrinsic molecular properties governing cellular uptake.

Since it is unknown whether the acyclic homologues of cyclotides can enter into cells and if so, via which endocytic routes, we focused our attention on a member of the squash trypsin inhibitor family, *Ecballium elaterium* trypsin inhibitor II (EETI-II), and explored its route of internalization. EETI-II is well characterized structurally and functionally, and has been shown to adopt a cystine-knot conformation but, unlike cyclotides, lacks a cyclic polypeptide backbone (Fig. 1a)^21^,^22^. Several attempts at biosynthesis of recombinant EETI-II in *E. coli* have been reported^22^,^23^,^24^. These methods rely on expression of the molecule of interest fused to a carrier protein that routes its secretion to the periplasmic space (oxidizing conditions) or to inclusion bodies (reducing conditions) which then requires additional folding steps after purification. Both of these strategies require many purification steps and do not generate high yields. Therefore, we first sought to develop a protocol for the biosynthesis of EETI-II in *E. coli* with good yields and purity that would be adaptable to allow for the future generation of cystine-knot peptide variants. Here, we also report that EETI-II is efficiently internalized into mammalian cells. Utilizing a fluorescently-labeled version of recombinant EETI-II, we demonstrate that EETI-II follows, in part, a macropinocytosis and clathrin-mediated endocytic route, is targeted to early endosomes, and accumulates in late endosome and lysosome compartments at later time points. Finally, using cell-penetrating peptides, we demonstrate the feasibility of enhancing the cellular uptake of EETI-II and modulating its trafficking in mammalian cells.

## Results

### Recombinant synthesis of EETI-II

**Figure 1:**
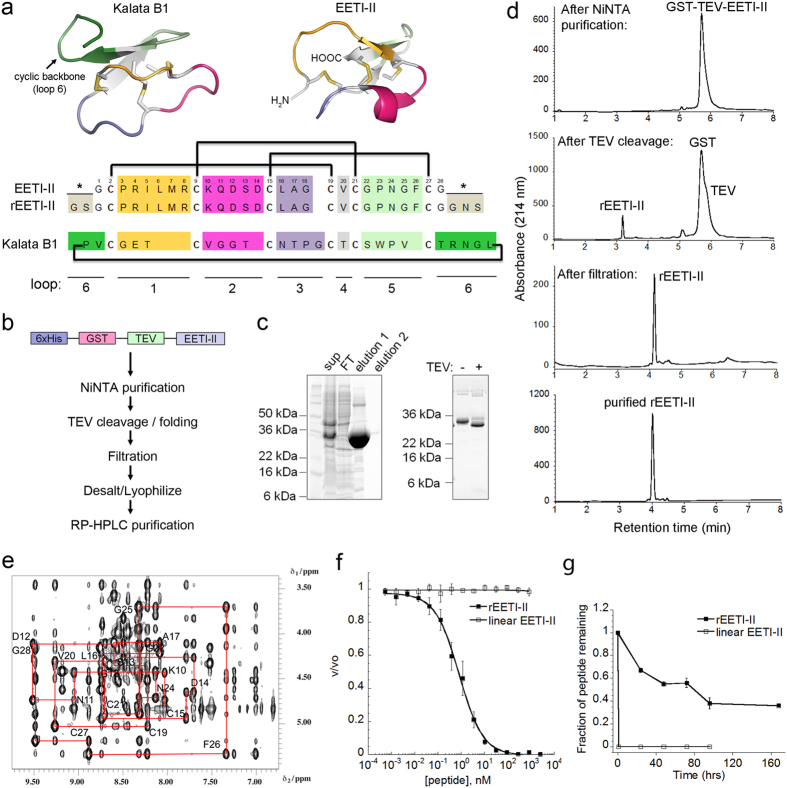
Generation and initial characterization of recombinant EETI-II. (**a**) 3D structures of the cyclotide kalata B1 and acyclic cystine-knot peptide EETI-II. Sequence alignment of wild type EETI, recombinant EETI-II (rEETI-II) and kalata B1 is shown. rEETI-II contains 2 non-native amino acids at the N-terminus and 3 non-native amino acids at the C-terminus (indicated by asterisks) due to TEV cleavage and cloning requirements, respectively. The sequence of the prototypic cyclotide kalata B1 is shown and loop 6 residues are highlighted in green. Cartoon representations for EETI-II (PDB entry 2IT7) and kalata B1 (PDB entry NB1) are shown. Colors represent the different structural loops (unrelated to those in Fig. 1b). (**b**) Strategy for generation of rEETI-II. (**c**) SDS-PAGE and (**d**) RP-HPLC analysis of the different stages of purification and TEV cleavage of rEETI-II fusion construct. Sup, supernatant; FT, flow-through; elution 1 and 2, elution from Ni-NTA column. In panel (**d**), the difference in retention time for rEETI-II post the TEV cleavage and filtration steps is due to change in buffer composition between the two samples. (**e**) Fingerprint region showing the amide to α-proton correlations in the 2D 1H-1H NOESY spectrum of rEETI-II. The freeze dried peptide was dissolved at a concentration of 3 mM in 180 μl 5% D_2_0/95% H_2_0, pH 2.2. Spectra were recorded at 600 MHz proton frequency at 298 K and with a mixing time of 300 ms. A sequential walk from residues 11 to 28 is shown. (**f**) rEETI-II inhibits trypsin enzymatic activity. A reduced linear version of EETI-II (linear-EETI) was used as a control. v/v0 is normalized proteolytic activity. (**g**) rEETI-II exhibits chemical stability in simulated gastric fluid at 37 °C compared to linear control.

Because of the moderate to low reported yields of EETI-II^23^, we first sought to improve the production process. Our strategy for the expression of recombinant EETI-II (rEETI-II) was to produce a version of EETI-II that is N-terminally fused with histidine (6xHis) and glutathione S-transferase (GST) tags in order to utilize nickel nitrilotriacetic acid (Ni-NTA) affinity chromatography for purification and use GST as a carrier protein to drive high expression and improve solubility (Fig. 1b). A Tobacco Etch Virus (TEV) protease cleavage site was inserted between the GST tag and EETI-II, allowing for the release of EETI-II from the fusion protein upon proteolytic processing. The cloning of this fusion construct resulted in several extra residues being incorporated into the final rEETI-II molecule, specifically Gly-Ser at the N terminus and Gly-Asn-Ser at the C-terminus (Fig. 1a). After confirming high expression of the fusion protein (30–40 mg/L) from cells grown in 1 L ultra-yield shake flasks, we scaled up *E. coli* growth to 10 L fermentation. On average, 1,500 mg of fusion protein was purified by Ni-NTA purification from 225 g of wet cell paste (Fig. 1c). The TEV cleavage reaction was conducted overnight in the presence of 3 mM reduced glutathione and 0.3 mM oxidized glutathione to provide a subtle redox buffer to support optimal TEV activity without reducing the disulfide bonds. The reaction was then filtered through an Amicon Stirred Cell device with a 10 kDa molecular weight cutoff, which effectively separated the folded peptide from the TEV protein and His-GST tags. A final reversed-phase high performance liquid chromatography (RP-HPLC) purification step was employed to isolate rEETI-II, and LC-MS was used to confirm the identity and high purity (>95%) of the peptide (Fig. 1d). The m/z data corresponded to the expected molecular weight of the fully oxidized peptide, confirming the formation of three disulfide bonds (Supplementary Fig. 1). The average overall yield of pure rEETI-II was 14 mg from 100 g of crude cell paste (five replicates). In sum, the method reported here simplifies the steps required to obtain pure and folded EETI-II in high yields.

### Structural characterization of rEETI-II

NMR was used to confirm the folding and disulfide bonding pattern in the solution structure of rEETI-II. NMR NOESY spectra of rEETI-II were measured using standard parameters and compared with the NOESY data back-calculated based on the coordinates and assignments of the published EETI-II structure^25^ (Fig. 1e and Supplementary Fig. 2). The overall fold was confirmed by comparison of the region containing the αH-βH NOESY cross peaks. Apart from few ambiguities due to peak overlaps and extra peaks in the measured data, which correspond to the additional amino acids present in rEETI-II, there is excellent agreement between the calculated and measured data (Supplementary Fig. 2a). Furthermore, to confirm the disulfide bond connectivity within rEETI-II, the distances of proton-proton (Hβ-Hβ) contacts across the disulfide bonds within the published structure (PDB entry 2IT7) were compared to those observed in the measured spectra of rEETI-II. The measured and back-calculated data show all of the expected cross peaks, confirming the C1–C4, C2–C5, C3–C6 disulfide bond connectivity (Supplementary Fig. 2b,c). Overall, these results indicate that rEETI-II generated via recombinant synthesis exhibits a cystine-knot fold similar to that of the native molecule in aqueous solution. Finally, this approach of generic analysis of focused inter-proton distances seems a fast and reliable method to confirm a peptide fold in multiple samples without having to perform a structure determination in each case.

### Functional and stability evaluation of rEETI-II

In order to further characterize the properties of the newly generated rEETI-II, we first assessed its ability to inhibit trypsin proteolytic activity. The native trypsin inhibitory activity of EETI-II is dependent on a correct cystine-knot topology, and hence its bioactivity serves as an indication of its proper folding^26^. A trypsin enzymatic assay showed that rEETI-II inhibited trypsin activity in cleaving a peptide substrate conjugated to AMC fluorophore (Arg-AMC), with a *K*_i_^app^ of 0.16 ± 0.01 nM (Fig. 1f). In contrast, a control reduced linear EETI-II, obtained via peptide synthesis and containing acetamidomethyl (Acm) protecting groups on all cysteine residues to prevent spontaneous disulfide bond formation, showed no effect on trypsin activity as expected, further validating the notion that folding is vital for the inhibitory activity of EETI-II.

We next evaluated the stability of folded rEETI-II relative to linear EETI-II control peptide. We found that rEETI-II was more stable in simulated gastric fluid (SGF) at 37 °C (50% remaining after several days) relative to its reduced counterpart, which was fully degraded within one hour (Fig. 1g). These results demonstrate that rEETI-II generated via the above recombinant synthesis approach is extremely stable under harsh conditions, as expected for a cystine-knot peptide.

### rEETI-II is rapidly internalized into mammalian cells via a membrane-dependent endocytic route

To investigate whether the acyclic cystine-knot peptide EETI-II is internalized on its own into cells, we first labeled purified rEETI-II with Alexa488 fluorescent dye using N-hydroxysuccinimidyl ester chemistry. The reaction product was purified by RP-HPLC and its identity as well as the folding of rEETI-II-A488 was confirmed by mass spectrometry (Supplementary Fig. 3). Moreover, rEETI-II-A488 inhibited trypsin activity, albeit with slightly reduced potency compared to unconjugated rEETI-II (*K*_i_^app^ of 0.71 ± 0.06 nM for rEETI-II-A488 vs. ~0.16 ± 0.01 nM for rEETI-II, Supplementary Fig. 4). Thus, labeling at the free amino groups (N-terminus and ε-amine of Lys10) is not detrimental to activity, consistent with other studies showing that the framework is tolerant to amino acid substitutions^23^.

**Figure 2:**
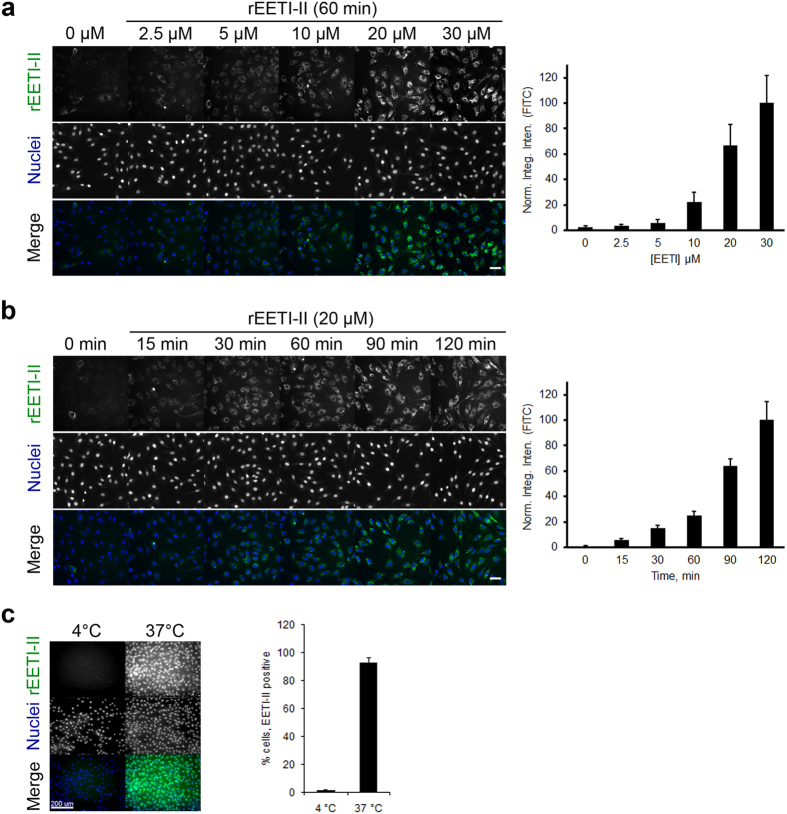
rEETI-II is internalized into mammalian cells. (**a,b**) Concentration- and time-dependent uptake of fluorescently-labeled rEETI-II. NIH-3T3 cells were treated with increasing concentrations of Alexa488-labeled rEETI-II for the indicated times. Cells were processed as described in methods. Scale bar, 50 μm. (**c**) Uptake of rEETI-II is temperature-dependent. NIH-3T3 cells were treated with rEETI-II-A488 (20 μM) for 1 hour at 37 °C or 4 °C. Scale bar, 200 μm. Cells were washed with PBS at the end of the study and fixed with 4% PFA. All samples were imaged on a high throughput ImageXpress Micro XL imaging system (Molecular Devices) with a 10x or 40x objective and images were analyzed by MetaXpress 4.0. Integrated fluorescence intensity values above a threshold defined using the DMSO-treated samples were measured and normalized to samples with the highest signal (panels a,b). Alternatively, percentage of rEETI-II-A488 positive cells with signal above background was measured (panel c). Mean ± SD. n = 1,400–1,500 cells per condition. Representative images from at least three independent experiments are shown.

Fluorescently-labeled rEETI-II-A488 was incubated for varying periods of time with different cells lines and its cellular uptake was monitored by fluorescence microscopy. We observed that rEETI-II-A488 was efficiently and rapidly internalized into mammalian cells in a concentration- and time-dependent manner (Fig. 2a,b). Interestingly, the uptake of rEETI-II-A488 appears to be independent of cell type as it was internalized equally well into both mouse fibroblast NIH-3T3 and HeLa cells (Fig. 2a,b and Supplementary Fig. 5), with greater than 80% of cells showing internalized rEETI-II-A488 positive signal above background after only 15 min incubation with 20 μM peptide. The intracellular distribution of internalized rEETI-II-A488 was similar in cells that have been fixed with paraformaldehyde versus live cells (*i.e.*, no fixation) (Supplementary Fig. 6), thereby ruling out potential artifacts that may impact subcellular distribution due to fixation as has been observed with other cell-penetrating peptides^27^. Moreover, cellular uptake of rEETI-II-A488 was completely abolished at 4 °C (Fig. 2c), indicating that the molecule is internalized via active endocytosis rather than a membrane translocation mechanism. It is noteworthy that little or no cell surface staining of rEETI-II-A488 was detected at 4 °C or 37 °C, suggesting minimal interaction with the membrane phospholipid bilayer; this phenotype is distinct from that observed with other cyclotides such as kalata B1^16^,^19^.

**Figure 3:**
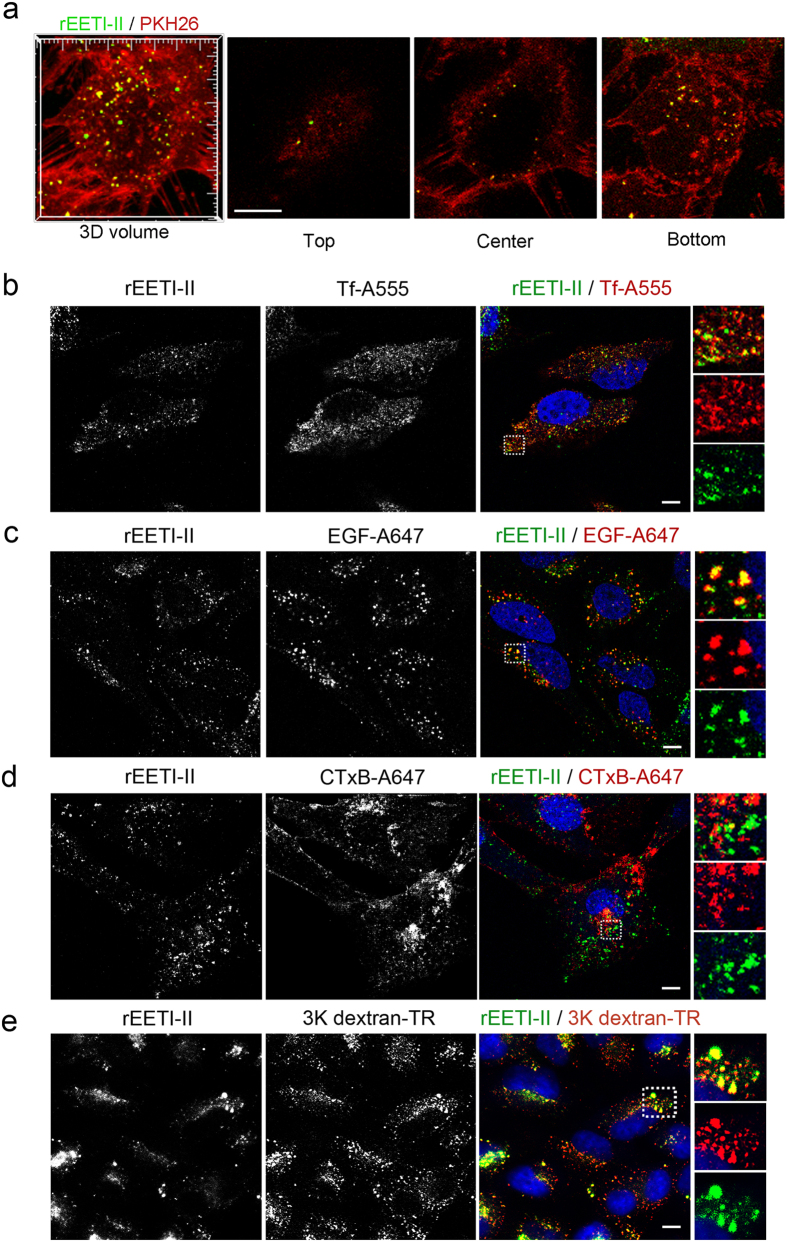
rEETI-II is internalized in part via a macropinocytosis and a clathrin-mediated endocytic pathway. (**a**) rEETI-II is internalized via membrane-bound vesicles that are derived from the plasma membrane. HeLa cells were labeled with the membrane marker PKH26 (4 μM) for 4 min, washed extensively with complete media and then incubated with rEETI-II-A488 (20 μM) for 45 min at 37 °C. Media were aspirated and the cells were fixed with 4% PFA and then imaged by fluorescence microscopy. Shown are the top, center and bottom planes of a representative 3D cell volume. Scale bar, 10 μm. Representative images from two independent experiments are shown. (**b**) rEETI-II-A488 is co-localized with transferrin-positive (Tf-A555, shown in red) vesicles in the early/recycling endosomes. (**c**) rEETI-II-A488 co-localizes with EGF-positive puncta (EGF-A647, shown in red, false colored). (**d**) rEETI-II-A488 does not co-localize with internalized cholera toxin (CTxB-A647, shown in red, false colored). HeLa (panels a–c) or NIH 3T3 (panel d) cells were incubated with rEETI-II-A488 (30 μM, 60 min) at 37 °C and endosomal markers (Tf-A555, 0.2 mg/ml; EGF-A647, 5 μg/ml; CTxB-A647, 25 μg/ml) were added in the last 10 min of incubation (the short incubation with transferrin and EFG was used to mainly target the probes to early endosome compartments). In panels b–d, cells were fixed with 4% PFA and processed as described in methods. Fluorescence images were captured on an upright LEICA SPE laser scanning confocal microscope (Leica Microsystems). Representative images from at least three independent experiments are shown. Scale bar, 15 μm. (**e**) rEETI-II-A488 (30 μM, 1 h) co-localizes with internalized 3 K dextran-TR (Texas red-conjugated, 0.1 mg/ml, 1 h, shown in red). HeLa cells were fixed with 4% PFA and processed as described in methods. Fluorescence images were captured on a high throughput ImageXpress Micro XL imaging system (Molecular Devices). Representative images from at least two independent experiments are shown. Scale bar, 10 μm.

To corroborate the above findings, we tracked the uptake of rEETI-II-A488 after *in situ* labeling of cell membranes with PKH26, a pH-independent yellow-orange fluorescent dye with long aliphatic tails that incorporates into the lipid regions of the plasma membrane by selective partitioning^28^,^29^,^30^. Adherent HeLa cells were treated with PKH26 dye for a brief period of time, quickly washed, and then rEETI-II-A488 was added to cells and its internalization was immediately followed over time. As PKH26-positive vesicles started to pinch off from the plasma membrane, internalized rEETI-II-A488 was found exclusively in membrane-bound intracellular vesicles that were labeled with PKH26 dye (Fig. 3a). Collectively, the above results indicate that rEETI-II is internalized via a membrane-dependent endocytic route that does not seem to involve plasma membrane transduction.

### Cellular uptake of rEETI-II involves in part a clathrin-mediated and macropinocytic pathway

To further characterize the endocytic pathway of rEETI-II, we co-incubated HeLa cells with rEETI-II-A488 and either Alexa555-conjugated transferrin (Tf-A555), Alexa647-conjugated epidermal growth factor (EGF-A647) or Alexa647-conjugated cholera toxin subunit B (CTxB-A647) and then monitored their cellular uptake. Internalized rEETI-II-A488 co-localized with internalized transferrin ligand and followed a similar endocytic route, indicating that it is targeted to the early/recycling endosome via a clathrin-mediated pathway (Fig. 3b). Consistent with this cellular uptake mechanism, we also observed co-localization of rEET-II-A488 with internalized EGF after 10 min, further validating the notion that rEET-II-A488 is being targeted to the endosomal system (Fig. 3c). On the other hand, there was little or no co-localization of rEET-II-A488 with internalized cholera toxin (Fig. 3d), which is typically endocytosed via a caveolin-dependent pathway. Internalized rEET-II-A488 was also found co-localized with 3 kDa Texas red-conjugated dextran, a marker of fluid phase uptake (Fig. 3e), suggesting that its uptake may also involve in part a macropinocytic pathway.

**Figure 4:**
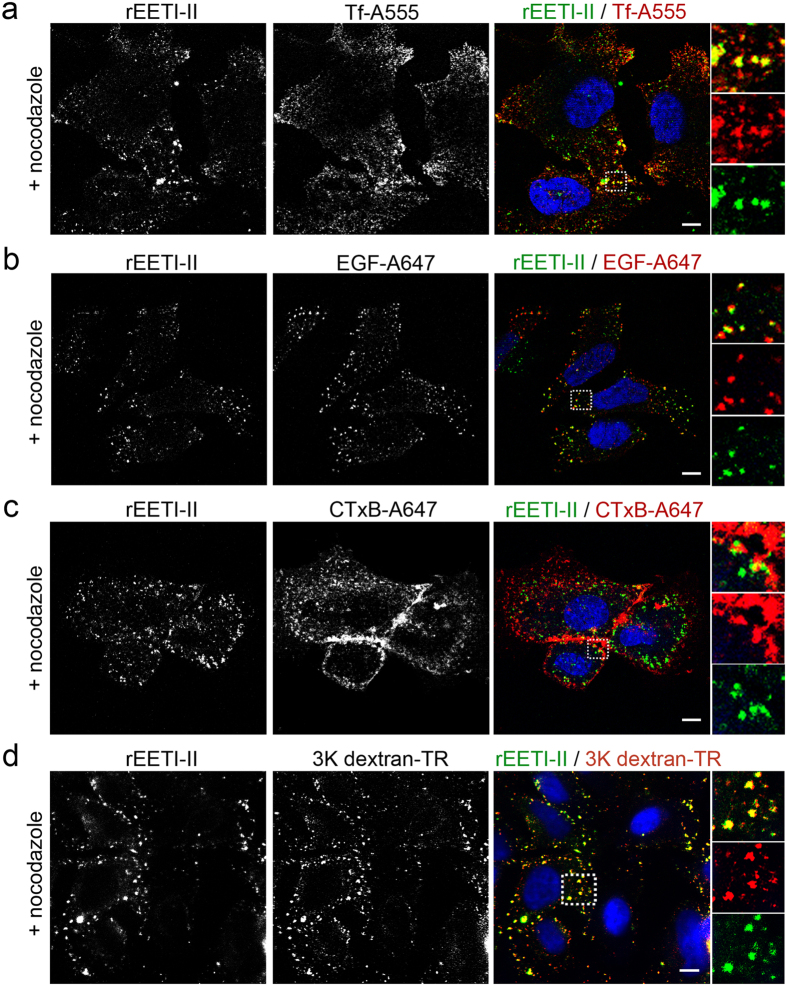
Post endocytic trafficking of rEETI-II is dependent on intact microtubules. (**a–d**) Treatment with nocodazole (microtubule polymerization inhibitor) alters the cellular distribution of internalized rEET-II-A488, transferrin, EGF and 3 K dextran. rEETI-II-A488 is co-localized with transferrin (**a**), EGF (**b**), 3 K dextran (**d**) but not cholera toxin subunit B (**c**), in the presence of the nocodazole. HeLa (panels a,b,d) or NIH 3T3 (panel c) cells were treated with nocodazole (10 μM) for 30 min then with rEETI-II-A488 (30 μM) for 60 min in the presence of nocodazole (10 μM) at 37 °C, with endosomal markers (Tf-A555, 0.2 mg/ml; EGF-A647, 5 μg/ml; CTxB-A647, 25 μg/ml) were added in the last 10 min of incubation. 3 K Dextran-TR (0.1 mg/ml) was added with rEETI-II-A488 at the same time to cells for 60 min. Cells were then fixed with 4% PFA and processed as described in methods. In panels a–c, fluorescence images were captured on an upright LEICA SPE laser scanning confocal microscope (Leica Microsystems). Representative images from at least three independent experiments are shown. Scale bar, 15 μm. In panel d, fluorescence images were captured on a high throughput ImageXpress Micro XL imaging system (Molecular Devices). Representative images from at least two independent experiments are shown. Scale bar, 10 μm.

Treatment with nocodazole, an inhibitor of microtubule polymerization which disrupts early endosome to late endosome trafficking but not transport from the plasma membrane to early endosomes^31^,^32^, altered the post-endocytic distribution of rEETI-II-A488 (Fig. 4 and Supplementary Figs 7–10), in agreement with the above proposed cellular uptake mechanism. In a control experiment, nocodazole also changed the distribution of internalized transferrin and EGF ligands, as well as 3 K dextran, from the perinuclear region to the cell periphery as expected^31^ and interestingly, rEETI-II-A488 appeared to co-localize with internalized transferrin and EGF in early endosomes at the cell periphery (Fig. 4). These findings indicate that internalized rEETI-II-A488 is being retained in early endosomes, along with transferrin and EGF, upon treatment with nocodazole, and reveal that transport of rEETI-II-A488 from early endosomes to late endosomes is dependent on microtubules.

### Internalized rEETI-II is targeted to early/recycling endosomes and subsequently accumulates in lysosomes

**Figure 5:**
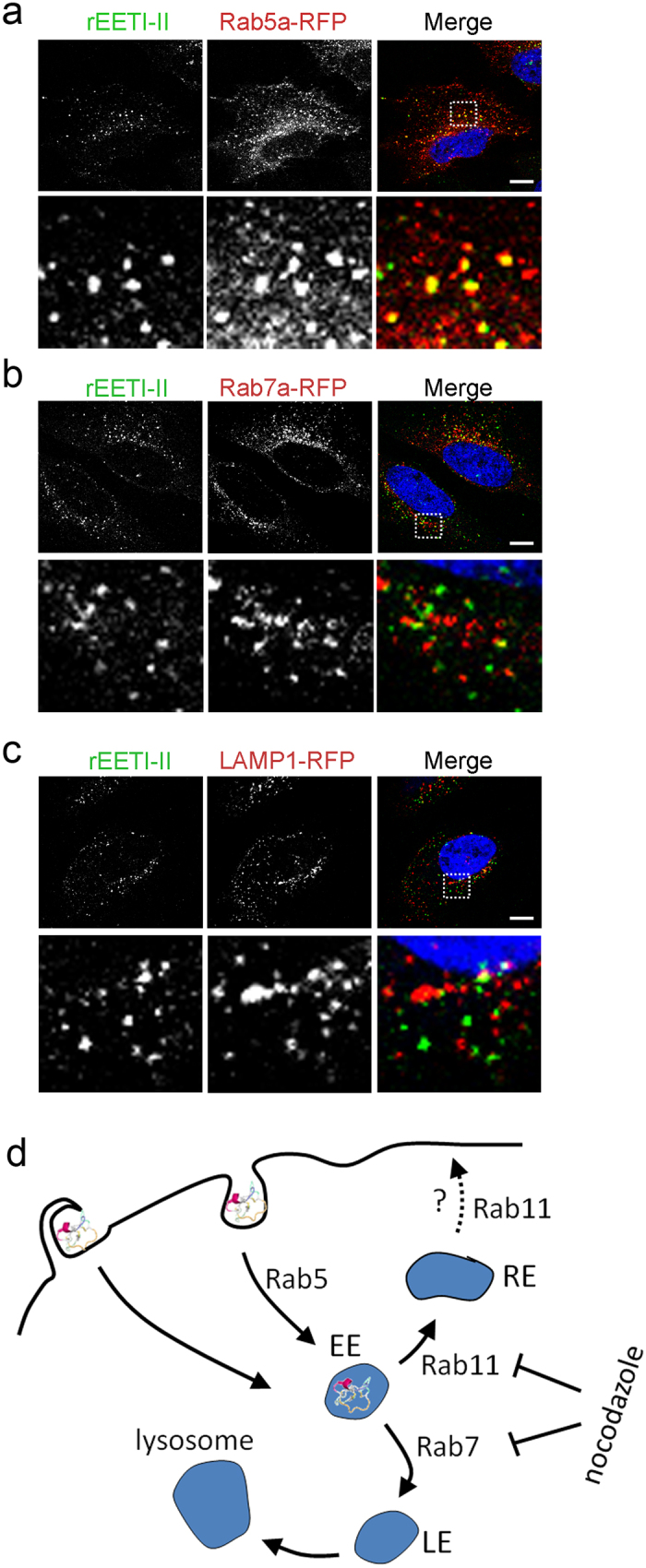
rEETI-II is initially internalized to early endosomes. (**a–c**) rEETI-II-A488 co-localizes with Rab5a (early endosome marker), but not Rab7a (late endosome marker) or LAMP1 (lysosome marker). HeLa cells over expressing Rab5a-RFP, Rab7a-RFP or LAMP1-RFP (all shown in red) were incubated with rEETI-II-A488 (30 μM, shown in green) for 1 h at 37 °C, then fixed with 4% PFA and processed as described in methods. Fluorescence images were captured on an upright LEICA SPE laser scanning confocal microscope (Leica Microsystems). Representative images from at least three independent experiments are shown. Scale bar, 10 μm. (**d**) Proposed model for endocytosis of EETI-II. EETI-II is internalized via an active endocytic pathway which is dependent on clathrin and subsequently microtubules, a mechanism that seems similar to that of transferrin and EGF at early time points. The uptake mechanism of EETI-II also involves macropinocytosis. Internalized EETI-II is initially targeted to Rab5a- and, at later time points, to Rab7a-positive endosomes and LAMP1-positive lysosomes. We propose that a subpopulation of EETI-II may potentially be recycled through Rab11 during the early phase of uptake. EE, early endosome; RE, recycling endosome; LE, late endosome.

To further characterize the identity of the endocytic compartment(s) to which rEET-II is targeted, HeLa cells were infected with baculoviruses encoding Rab5a and Rab7a, markers of the early and late endosomes, respectively. Upon continuous incubation with rEETI-II-A488 for 60 min, followed by fixation and confocal microscopy imaging, rEETI-II-A488 was found primarily localized with Rab5a-positive vesicles, but to a much lesser extent with Rab7a-positive puncta (Fig. 5a,b), suggesting that EETI-II is initially targeted to the early endosome/recycling pathway. Consistent with this notion, rEETI-II-A488 showed minimal colocalization with the lysosome marker LAMP1 after a 60 min incubation (Fig. 5c). However, at later time points (3, 6 and 24 h), rEETI-II-A488 started to appear gradually in late endosomes and lysosomes (Supplementary Fig. 11), with its fluorescence signal dramatically increasing in lysosomes after 24 h.

The above findings were further corroborated by pulse chase experiments. In cells that were treated with rEETI-II-A488 for 3 hours and then chased with medium over a 24-hour period, we observed a time-dependent clearance of rEETI-II-A488 from Rab5a-positive endosomes, which was concomitant with its accumulation in lysosomes over time (Supplementary Fig. 12). It is possible that recycling of internalized rEETI-II-A488 also occurred to some degree at earlier time points as suggested by its 3-hour colocalization pattern with internalized transferrin ligand (Supplementary Fig. 13), which is targeted to the recycling endosome within this time frame. These data suggest a possible Rab11-dependent process for rEETI-II recycling. In sum, our findings support a model in which EETI-II is uptaken into cells in part via a clathrin-mediated and macropinocytosis endocytic route which targets it to early endosomes, subsequently accumulating in lysosomes (Fig. 5d).

### Trafficking and subcellular distribution of rEETI-II is altered upon co-incubation with a cell-penetrating peptide

To explore the feasibility of re-targeting rEETI-II to other cellular compartments outside the endosomal system, we utilized a cell-penetrating peptide Xfect that had been reported to form complexes with large proteins such as bovine serum albumin and facilitate its uptake into human hepatoma multicellular tumor spheroids^33^. Although the mechanism of cellular internalization of Xfect is largely unknown, it seems to utilize caveolin-dependent and macropinocytic pathways^33^. Since it is also unclear whether Xfect is compatible with and enables cellular uptake of small-size peptides, we first sought to study the cellular uptake of rEETI-II in the presence of Xfect.

**Figure 6:**
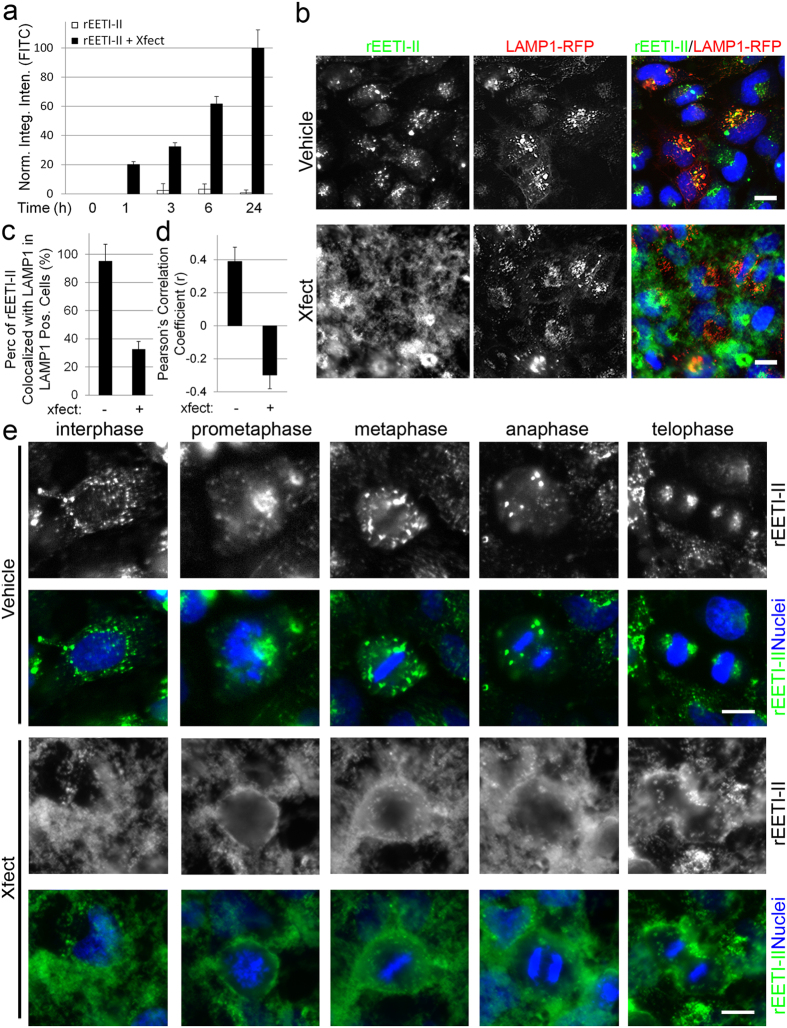
A cell-penetrating peptide enhances cellular uptake of rEETI-II and alters its subcellular distribution pattern. (**a**) Co-incubation of rEETI-II-A488 (1 μM) with Xfect (Xfect: rEETI-II-A488 = 4:1) enhances cellular uptake of rEETI-II. HeLa cells were treated with rEETI-II-A488/Xfect complexes or rEETI-II-A488 alone for the indicated times, then washed and fixed with 4% PFA as described in methods. Fluorescence images were captured on a high throughput ImageXpress Micro XL imaging system (Molecular Devices) with a 40x objective and images were analyzed by MetaXpress 4.0 (for images, see Supplementary Fig. 14). Integrated fluorescence intensity values above a threshold defined using the DMSO-treated samples were measured and normalized to samples with the highest signal (24 h incubation with the rEETI-II-A488/Xfect complexes). Mean ± SD. n = 1,000 cells. (**b**) Co-incubation of rEETI-II-A488 (5 μM) with Xfect (Xfect: rEETI-II-A488 = 4:1; 6 h incubation) alters its subcellular distribution, leading to minimal colocalization with lysosomes. HeLa cells were transduced with LAMP1-RFP for 24 h then treated with rEETI-II-A488/Xfect complexes or rEETI-II-A488 alone for 6 h. Cells were washed and fixed with 4% PFA as described in methods. Fluorescence images were captured on a high throughput ImageXpress Micro XL imaging system (Molecular Devices) with a 40x objective. Representative images from four independent experiments are shown. (**c**) Colocalization of rEETI-II-A488 and LAMP1-RFP was analyzed by MetaXpress 4.0. Percentage of rEETI-II-A488 colocalized with LAMP1-RFP (above a threshold defined using the DMSO-treated samples) was measured and normalized to the percentage of LAMP1-RFP positive cells. Mean ± SD. n = 1,700 cells. (**d**) Colocalization of rEETI-II-A488 and LAMP1-RFP was measured using Pearson’s correlation coefficient (r) in ImageJ (Fiji). Mean ± SD. n = 100 cells. (**e**) Xfect alters the punctate distribution of rEETI-II-A488 throughout different stages of cell division. Due to the low fluorescence signal, images for cellular samples treated with rEETI-II-A488 only were adjusted for brightness independently in order to show the cellular distribution of rEETI-II-A488. Representative images from four independent experiments are shown. Scale bar: 20 μm.

Co-incubation of Xfect with rEETI-II-A488 substantially increased the cellular uptake of the latter and dramatically altered its subcellular distribution (Fig. 6a,b and Supplementary Figs 14 and 15). Strikingly, after a 6-hour uptake, internalized rEETI-II-A488 delivered via Xfect exhibited a diffused and more spread fluorescence pattern across subcellular regions in comparison to the strictly punctate pattern observed with rEETI-II-A488 treatment only (Fig. 6b and Supplementary Fig. 16). It also demonstrated minimal colocalization and a negative Pearson’s correlation with LAMP1 (Fig. 6b–d), indicating that treatment with Xfect alters the trafficking of rEETI-II-A488 to predominantly evade lysosomes. In contrast, substantial colocalization of internalized rEETI-II-A488 was observed with LAMP1 in the absence of Xfect (Fig. 6b–d). It is noteworthy that no cellular toxicity, as determined by nuclear morphology, was observed in the Xfect-treated samples.

To further understand the scope of internalization of rEETI-II in the presence or absence of Xfect, we monitored its uptake in dividing cells. Internalized rEETI-II-A488 exhibited a punctate pattern during different stages of cell division, consistent with its cellular uptake (Fig. 6e). This phenotype changed from punctate to a more dispersed pattern in dividing cells incubated with both rEETI-II-A488 and Xfect (see metaphase and anaphase, Fig. 6e), suggesting that the internalization and trafficking of EETI-II could also be modulated in dividing cells.

The striking and unexpected changes in the cellular uptake and distribution of rEETI-II observed with Xfect led us to investigate further its mechanism of internalization. Cellular uptake of rEETI-II-A488 co-incubated with Xfect was mostly blocked at 4 °C (Supplementary Fig. 17a). However, rEETI-II-A488 showed binding to the cell surface under these conditions, indicating that the rEETI-II/Xfect complex has a higher propensity to bind to cellular membranes compared to rEETI-II alone which did not show cell surface staining at 4 °C (Fig. 2c and Supplementary Fig. 17b). Additionally, the uptake of rEETI-II-A488/Xfect into different subcellular regions was time-dependent (Supplementary Fig. 18). Finally, permeabilization with 0.1% Triton X-100 did not affect the subcellular localization of internalized rEETI-II/Xfect, in contrast to rEETI-II alone (Supplementary Fig. 19), indicating that the internalized rEETI-II/Xfect material is bound to subcellular membranes, whose identity remains to be elucidated.

**Figure 7:**
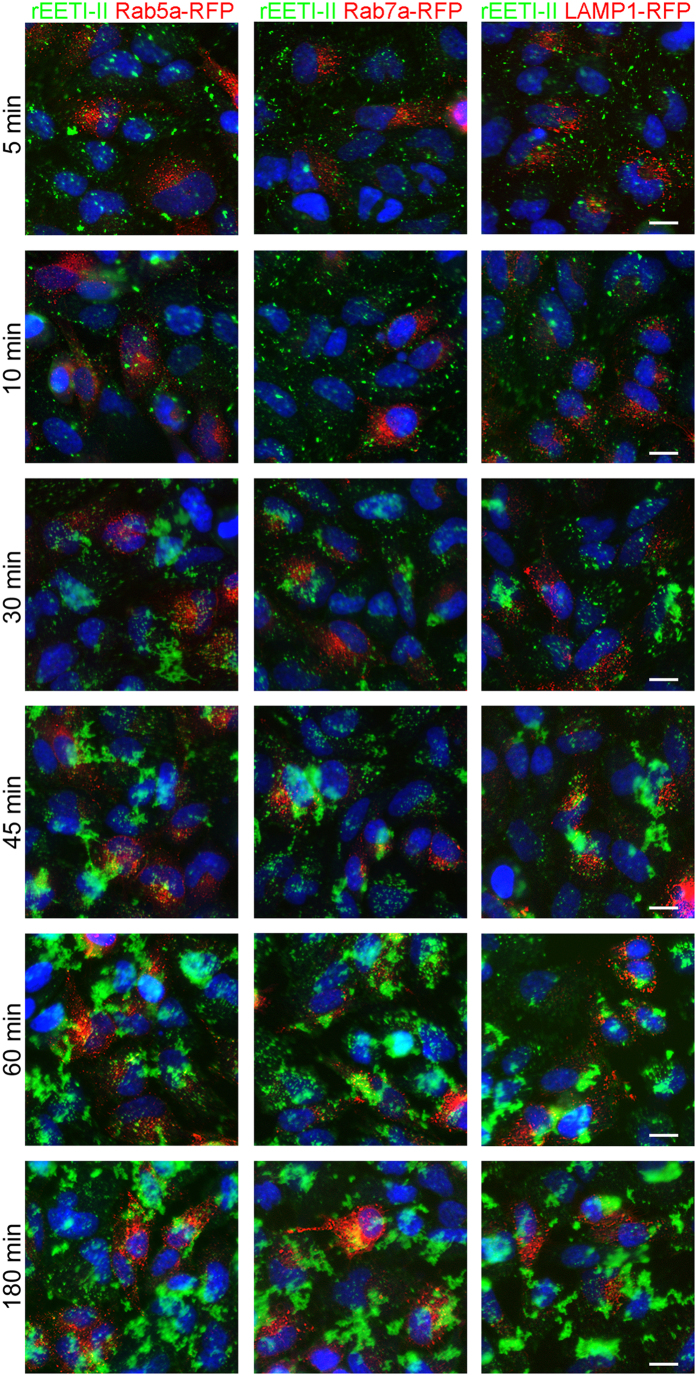
The peptide Xfect modulates the cellular uptake mechanism of rEETI-II. Co-incubation of rEETI-II-A488 (1 μM) with Xfect (Xfect: rEETI-II-A488 = 4:1) changes the subcellular distribution of rEETI-II at different time points. HeLa cells were transduced with Rab5a-RFP, Rab7a-RFP or LAMP1-RFP (shown in red) for 24 h and were then treated with rEETI-II-A488/Xfect (shown in green) for the indicated times at 37 °C, then washed and fixed with 4% PFA as described in methods. Fluorescence images were captured on a high throughput ImageXpress Micro XL imaging system (Molecular Devices) with a 40x objective Representative images from three independent experiments are shown. Scale bar, 20 μm. Images are adjusted differently at various time points to show the subcellular distribution of rEETI-II/Xfect during the early phase of uptake.

The above findings prompted us to investigate in more detail the subcellular distribution and route of entry of the rEETI-II/Xfect complex. HeLa cells were transduced with Rab5a-RFP, Rab7a-RFP or LAMP1-RFP for 24 h and were then treated with rEETI-II-A488/Xfect complexes or rEETI-II-A488 alone for different time points. The bulk of rEETI-II-A488 that was delivered by co-incubation with Xfect showed some co-localization with early (Rab5a-positive) endosomes at early time points within 1 h of uptake, but it exhibited little or minimal colocalization with late (Rab7a-positive) endosomes and lysosomes (LAMP1-positive) at later time points within the 3 h uptake period (Fig. 7). This altered subcellular distribution is in stark contrast to that observed for rEETI-II alone which showed a time-dependent predominant targeting to early and late endosomes and subsequently lysosomes, as described above (Fig. 5 and Supplementary Fig. 11). In sum, our data demonstrate that treatment with Xfect enhanced the cellular uptake of rEETI-II, and modulated its internalization and trafficking route, as exemplified by the drastic change in its subcellular distribution profile and diminished accumulation in lysosomes. The nature of the cellular compartment that rEETI-II is targeted to upon treatment with Xfect remains unclear.

## Discussion

Here, we describe a novel protocol for the efficient generation of recombinant folded EETI-II in high yields and to near homogeneity. The method can potentially be applied to generating other acyclic cystine-knot peptides and would find use in generating variants to study the molecular basis for EETI-II cellular uptake. Notably, our studies reveal that the acyclic cystine-knot EETI-II is endocytosed into cells, in part via clathrin-mediated and macropinocytosis pathways as supported by the following observations: (a) No uptake or significant cell surface binding is observed at 4 °C; (b) colocalization with Tf- and EGF-positive puncta but not with internalized cholera toxin; (c) colocalization with internalized 3 K dextran; (d) colocalization with Rab5a- but not Rab7a-positive endosomes or LAMP1-positive lysosomes at early time points; and (e) no post endocytic trafficking, as observed by re-distribution of internalized puncta to the cell periphery, in the presence of the microtubule inhibitor nocodazole. Our observation that EETI-II is colocalized with transferrin (3 hour treatment) at the recycling endosome suggests that a population of EETI-II may be recycled. However, the bulk of internalized EETI-II seems to be routed to late endosomes and eventually accumulates in lysosomes (Fig. 5d). Overall, our data reveal the potential of acyclic cystine-knot peptides for endocytic targeting.

The lack of cyclization, as exemplified here by rEETI-II, does not prevent its internalization compared to the cyclic counterparts such as MCoTI-II and kalata B1. However, quantitative studies comparing the uptake of cyclic and acyclic versions would be needed to delineate any subtle differences in the uptake mechanism. Clearly, other physical properties of the molecules would likely play an important role in their endocytosis. We propose that the rigidity of the framework, coupled with its solvent-exposed hydrophobic residues, likely contribute to its ability to be uptaken into cells. Additionally, the presence of a combination of basic and hydrophobic residues within EETI-II might also influence its cellular internalization, and these contributions would need to be carefully studied in a systematic manner on a case-by-case basis. It also remains an open question whether cyclic and acyclic cystine-knot peptides can be leveraged to target areas in the cell outside the endosomal compartments. Our proof-of-concept experiments with Xfect demonstrate the feasibility for enhancing the cellular uptake and modulating the subcellular distribution of rEETI-II, and suggest a potential path forward for delivering rEETI-II to different subcellular regions. Consistent with this notion, a modified version of the cyclic cystine-knot peptide MCoTI-I, which incorporates an alpha helix that targets p53, displayed on-target biological activity in cells^34^. Given that MCoTI is internalized into late endosomes and lysosomes and does not seem to escape the endosomal compartments^17^, it is intriguing how it exhibits activity against a cytosolic protein target such as p53. In this case, it is possible that the newly engineered molecule serves as a new entity that is capable of escaping endosomes, a mechanism that has not been established yet and is worth investigating in order to help delineate this disparity. Nonetheless, our findings establish for the first time that the trafficking of cystine-knot peptides could be re-routed inside cells.

In conclusion, we describe the uptake of EETI-II in interphase and mitotic cells and provide proof-of-concept studies for modulating its trafficking in order to diminish its accumulation in lysosomes. The findings in this study underscore the potential utility of EETI-II, and perhaps other cystine-knot peptides, as promising scaffolds for targeting intracellular organelles and proteins. Understanding the intrinsic molecular properties that mediate the cellular entry of the different cystine-knot peptides and how these influence their distinct cellular uptake mechanisms is of vital importance. This information should prove useful in the design and development of cell-permeable cystine-knot peptides. Furthermore, efforts to engineer strategies that enable escape from the endocytic compartment are desirable and our findings support progress in this direction in order to fully unlock the therapeutic potential of cystine-knot peptides in drug discovery and delivery.

## Methods

### Cloning

Genes encoding the EETI-II sequence were synthesized by GenScript and were designed to contain a BamHI restriction site at the N-terminus and an EcoRI restriction site at the C-terminus. The synthesized gene was subcloned into a modified pET-52b E. *coli* expression vector (Novagen) at BamHI/EcoRI restriction sites directly C-terminal to (and in frame with) a 6xHis-GST-TEV tag.

### Biosynthesis and purification of EETI-II

EETI-II was fused to an N-terminal His-GST tag and expressed as a soluble protein in *E. coli* using standard protocols. Proteins were expressed in 44H9 cells and grown in Complete C.R.A.P. media in a 10 L fermenter at 37 °C^14^. The cells were subsequently harvested by centrifugation and frozen at −80 °C. After thawing, the cells were resuspended in lysis buffer (50 mM Tris pH 8.0, 300 mM NaCl, 10 mM imidazole, 2 mM β-Mercaptoethanol (BME), 0.4% n-Dodecyl-β-D-Maltopyranoside (DDM), 5 U/ml Benzonase (Sigma #E1014), Roche Protease inhibitor tablets (#11 873 580 001)), and then passed through a microfluidizer (Microfluidics International Corporation) three times to ensure lysis. Insoluble debris was removed by centrifugation at 30,000 *g* for 30 min at 4 °C. The cleared supernatant was collected and filtered using a 0.22 μm Stericup^®^ (Millipore).

Recombinant GST-EETI-II was captured from the lysate by nickel-nitrilotriacetic acid (Ni-NTA) agarose (Qiagen). The resin was washed with 5 column volumes (CV) of wash buffer (50 mM Tris-HCl pH 8.0, 300 mM NaCl, 20 mM imidazole, 2 mM BME) and the fusion protein was eluted with 3–4 x CV of wash buffer containing 400 mM imidazole. The elution fractions were pooled and 250 μg Tobacco Etch Virus (TEV) protease was added per 20 mg of GST-EETI-II fusion protein. The TEV cleavage reaction was supplemented with 3 mM reduced glutathione and 0.3 mM oxidized glutathione followed by overnight incubation at 4 °C with gentle shaking. The rEETI-II was isolated by filtration (10 kDa cutoff filter, Amicon Stirred Cell Device, Millipore), which trapped the His-GST tag and TEV proteins while allowing rEETI-II to pass through the membrane, followed by de-salting on a C18 SepPak column (Waters #WAT043345). The final product was purified using RP-HPLC (Varian) on a C18 column (Higgins Analytical #RS-2520-W181) using a gradient of 2–50% acetonitrile over 40 min at a flow rate of 20 mL/min. The folded rEETI-II eluted as a sharp peak at 23 min and fractions were analyzed by analytical LC-MS (Agilent 1200 series) to confirm folding and purity and then were subsequently pooled.

### Trypsin enzymatic assay

Assays were done as described earlier with slight modification^14^. Briefly, normalized proteolytic activity of trypsin (2.3 pM) toward tosyl-GPR-AMC substrate (5 μM) was measured for 2 h after pre-incubation of the peptides with protease for 1 h at room temperature. The data were plotted and fitted using KaleidaGraph v3.6 software. Apparent substrate inhibition constant (Ki^app^) was determined from at least three independent measurements.

### NMR

^1^H NMR spectra were recorded on a Bruker AVANCE-600 spectrometer operating at a proton resonance frequency of 600 MHz. All two-dimensional spectra were collected in the phase sensitive mode using the TPPI (time proportional phase incrementation) method^35^. Spectra were recorded at 298 K and calibrated to the chemical shift of DSS at 0 ppm. NOESY^36^ two-dimensional spectra were recorded with 4 k data points in the acquisition dimension, 512 data points in the indirect dimension and a mixing time of 300 ms. The time-domain data were processed using the software TOPSPIN (Bruker, Karlsruhe); evaluation was performed using the program AURELIA^37^. The NMR structure was obtained from the Brookhaven Protein Database and visualized in PYMOL (The PyMOL Molecular Graphics System, Version 1.7.4 Schrödinger, LLC.).

### Back-calculation of NOESY spectra

NOESY spectra were back-calculated using the relaxation-matrix approach^38^ as implemented in the program RELAX^39^. The coordinates of the published EETI-II structure (PDB entry 2IT7) were used to back-calculate NOESY spectra based on the experimentally confirmed published assignments. These spectra were compared to experimental spectra. Specific distances were extracted from the structure and compared to the signal intensity in the experimental spectrum.

### Stability Assays

Peptides were incubated at a concentration of 0.3 mg/ml in simulated gastric fluid at 37 °C for the indicated times. To prepare simulated gastric fluid, 2 g of sodium chloride, 43 mg of sodium thaurocholate, 16 mg of lecithin and 100 mg of pepsin were dissolved in 1 L of Milli-Q water (18.2 MΩ.cm^−1^) and adjusted to pH 1.2 with 37% hydrochloric acid. At the indicated time points, 10 μl (3 μg) aliquots were removed from the reaction, quenched with 0.25 M NaOH and then mixed with 2.6 μg of the internal standard. Samples were immediately analyzed by analytical LC/MS (Agilent 1200 series) and the area under the A_214nm_ peak was quantified. Wild-type EETI-II generated by peptide synthesis was used as an internal reference for linear EETI-II (which contains acetamidomethyl (Acm) protecting groups on all cysteine residues) and rEETI-II samples.

### Fluorescence labeling of rEETI-II

Purified rEETI-II was labeled at free amines with NHS-AlexaFluor488 (Invitrogen, cat #A-20000) according to the manufacturer’s directions. All labeling reactions were done with a molar ratio of 1: 3 (rEETI-II: dye; 2 mg/ml rEETI-II) in 0.1 M sodium bicarbonate (pH 7.5) and incubated with gentle rocking at room temp for 18 hours in the dark. Excess free dye was removed by size exclusion chromatography using Bio-Gel P2 resins with a fractionation range of 100–800 Da (BioRad cat #150-4114). Labeled rEETI-II was lyophilized, reconstituted in 50% DMSO (in H_2_O) and purified with a C18 reversed phase HPLC column. The final product (rEETI-II-A488) was confirmed by mass spectrometry using a LC-MS system (Agilent Technologies) and comprises a mixture of single- and dual-labeled rEETI-II (at lysine K10 residue and the N-terminus).

### Cell culture

HeLa cells (ATCC no. CCL-2) or NIH 3T3 cells (ATCC no. CRL-1658) were grown in high glucose Dulbecco’s Modified Eagle’s Medium (DMEM) supplemented with 10% FBS and 2 mM Glutamax™. All cells were incubated in a 5% CO_2_ humidified incubator at 37 °C for 24 h before any experiments. Cells were seeded onto either 18 mm glass coverslips in a 12 well culture plate (300,000 cells per well), Corning CellBIND 96 well plates (10,000 cells per well) or μ-Plate 96 well ibiTreat plates (ibidi) (10,000 cells per well), and were grown for 24 h at 37 °C/5% CO_2_ before experiments.

### Fluorescence imaging of cellular uptake of rEETI-II-A488

NIH 3T3 or HeLa cells were grown on Corning CellBIND 96-well plates for 24 h at 37 °C/5% CO_2_ before experiments. The cells were incubated with rEETI-II-A488 containing medium (100 μl each well) at 37 °C/5% CO_2_ for the indicated times, washed twice with cold PBS, once with acid buffer (0.2 M Glycine, 0.15 M NaCl, pH 3.0), and twice with cold PBS. Cells were then fixed with 4% PFA at room temperature for 20 min, and washed three times with PBS. To stain nuclei, cells were incubated with Hoechst 333421 (5 μg/ml in PBS) for 10 min and washed three times with PBS then stored in 100 μl PBS in the dark until image acquisition. For live cell imaging, cells were washed three times with HBSS, incubated with Hoechst 333421 (5 μg/ml in HBSS) for 5 min at 37 °C and washed twice with HBSS then stored in 100 μl HBSS and imaged immediately. Fluorescence images for samples on Corning CellBIND 96-well plates were captured on a high throughput ImageXpress Micro XL imaging system (Molecular Devices) and images were analyzed by MetaXpress 4.0. Integrated fluorescence intensity values above a threshold defined using the DMSO-treated samples were measured and normalized to samples with the highest signal.

### Preparation of rEETI-II-A488/Xfect complex

rEETI-II-A488 were co-incubated with the cell-penetrating peptide (Xfect™ Protein Transfection Reagent; Clontech, cat #631324/rEETI-II-A488: Xfect; 1 μg: 4 μl) following the manufacturer’s protocol. Briefly, 16 μl of 1X Xfect protein transfection reagent stock solution (or deionized water only for control experiments with rEETI-II-A488 alone) were used to prepare complexes containing 4 μg of rEETI-II-A488 (final volume 100 μl; final concentration 10 μM). The complexes were incubated at room temperature for 30 min. To treat cells with complexes containing 5 μM rEETI-II-A488, 50 μl of the mixture was added to cells with 50 μl Opti-MEM. To treat cells with complexes containing 1 μM rEETI-II-A488, 10 μl of the mixture was added to cells with 90 μl Opti-MEM. Cells were incubated for the indicated times then processed for live or fixed cell imaging.

### Cell membrane labeling assay

*In situ* labeling of adherent cells with PKH26 (Sigma, cat #MINI26) was done according to manufacturer’s recommendations with modifications. Different concentrations of the labeling dye and different incubation times were tested to obtain optimal cell membrane staining. HeLa cells (60,000 cells per coverslip per well in a 12-well plate) were washed three times with serum-free medium, incubated with PKH26 (4 μM) for 4 min in serum-free medium, and then an equal volume of FBS was added to cells and allowed to incubate for an additional 1 min. After aspirating the media, cells were washed three times with complete media and then incubated immediately with rEETI-II-A488 (20 μM) for 45 min at 37 °C. Media were aspirated and the cells were fixed with 4% PFA for 20 min, washed with PBS and then imaged by confocal microscopy.

### Endocytosis assays and inhibitor treatment

NIH 3T3 or HeLa cells were pre-incubated with nocodazole (10 μM; Sigma, cat #M1404) containing medium for 30 min at 37 °C/5% CO_2_ (when necessary), then incubated with 30 μM rEETI-II-A488 for 60 min (in the presence or absence of nocodazole). Tf-A555 (0.2 mg/ml, Life Technologies, cat #T35352), EGF-A647 (5 μg/ml, Life Technologies, cat #E35351), or CTxB-A647 (25 μg/ml, Life Technologies, cat #C34778) were added in the last 10 min of incubation. Texas Red-dextran (3,000 MW, lysine fixable, 0.1 mg/ml, Life Technologies, cat #D3328) was added together with rEETI-II-A488 (60 min incubation).

To label recycling endosomes, cells were incubated with rEETI-A488 (30 μM) and Tf-A555 (0.2 mg/ml) for 3 h. For transduction with CellLight^®^ BacMam 2.0 reagents (Rab5a-RFP, Rab7a-RFP or LAMP1-RFP; Life Technologies), HeLa cells plated on coverslips were incubated with 0.5 ml medium containing 50 μl CellLight^®^ BacMam 2.0 reagent for 24 h (or 150 μl medium containing 15 μl CellLight^®^ BacMam 2.0 reagent for cells plated on 96 well plates). After removal of the medium, cells were washed with fresh medium twice and incubated with rEETI-II-A488 (10 or 30 μM) for the indicated times. All cells were washed twice with cold PBS, once with acid buffer (0.2 M Glycine, 0.15 M NaCl, pH 3.0), and twice with cold PBS. Cells were then fixed with 4% PFA at room temperature for 20 min, and washed three times with PBS. The coverslips were mounted with Prolong Gold Antifade Mountant with DAPI (Life Technologies, cat #P-36931) and protected from light until image acquisition. For samples on 96-well plates, cells were incubated with Hoechst 333421 (5 μg/ml in PBS) for 10 min and washed three times with PBS then stored in 100 μl PBS until image acquisition. Fluorescence images for samples on Corning CellBIND 96-well plates were acquired on ImageXpress Micro XL imaging system (Molecular Devices). All samples were protected from light.

### Confocal microscopy

Fluorescence images for samples on coverslips were captured on an upright LEICA SPE (or an inverted LEICA SP5 for μ-Plate 96 Well ibiTreat plates (ibidi)) laser scanning confocal microscope (Leica Microsystems). Images were processed and analyzed with LAS AF image software (Leica Microsystems).

## Additional Information

**How to cite this article**: Gao, X. *et al*. Cellular uptake of a cystine-knot peptide and modulation of its intracellular trafficking. *Sci. Rep.* **6**, 35179; doi: 10.1038/srep35179 (2016).

## Supplementary Material

Supplementary Information
