# Supplementary material for: Cellular uptake of a cystine-knot peptide and modulation of its intracellular trafficking
Source: Sci Rep. 2016 Oct 13;6:35179. doi: 10.1038/srep35179 (PMC5062073; doi:10.1038/srep35179)
Supplement: Supplementary Information [file srep35179-s1.pdf]

# Supporting Information

**Cellular uptake of a cystine-knot peptide and modulation of its intracellular trafficking**

Xinxin Gao, Karen Stanger, Harini Kaluarachchi, Till Maurer, Paulina Ciepla, Cecile Chalouni, Yvonne Franke and Rami N. Hannoush*

*Departments of Early Discovery Biochemistry, Structural Biology and Pathology, Genentech, 1 DNA Way, South San Francisco, California*

* To whom correspondence should be addressed: hannoush.rami@gene.com

a

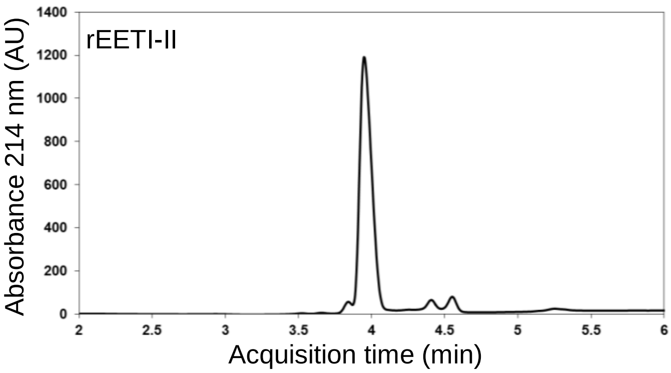


b

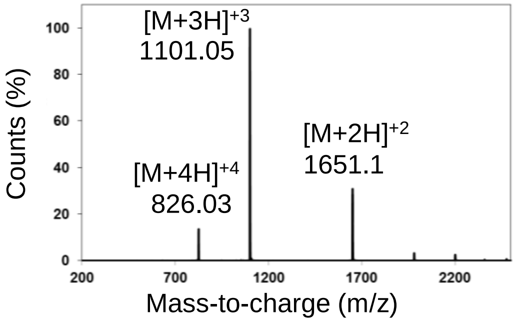


c

| **Ion species** | **[M+2H]$^{+2}$** | **[M+3H]$^{+3}$** | **[M+4H]$^{+4}$** |
|---|---|---|---|
| **Expected MW** | **1651.4** | **1101.27** | **826.2** |
| **Observed MW** | **1651.1** | **1101.05** | **826.03** |

**Supplementary Figure 1. LC-MS analysis of purified folded rEETI-II.** Representative LC trace (a) and mass spectrometry analysis (b,c) of rEETI-II after RP-HPLC purification as described in methods.

a

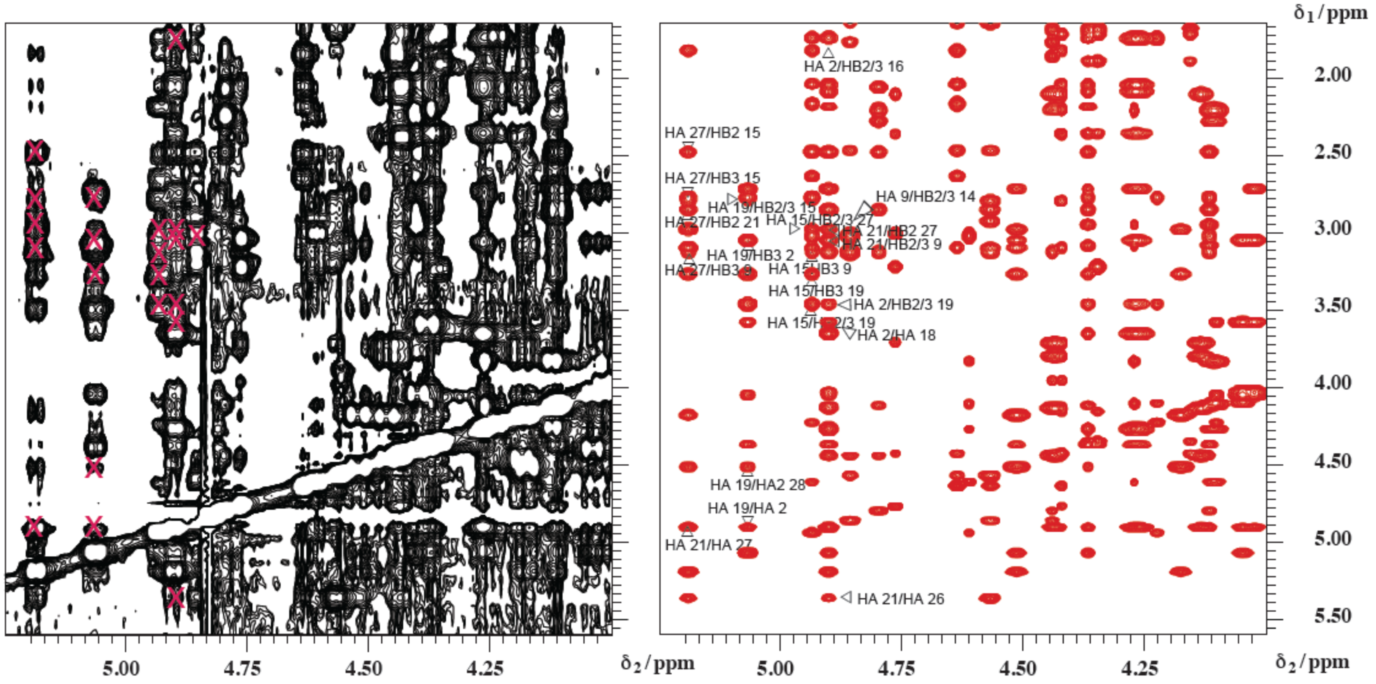


b

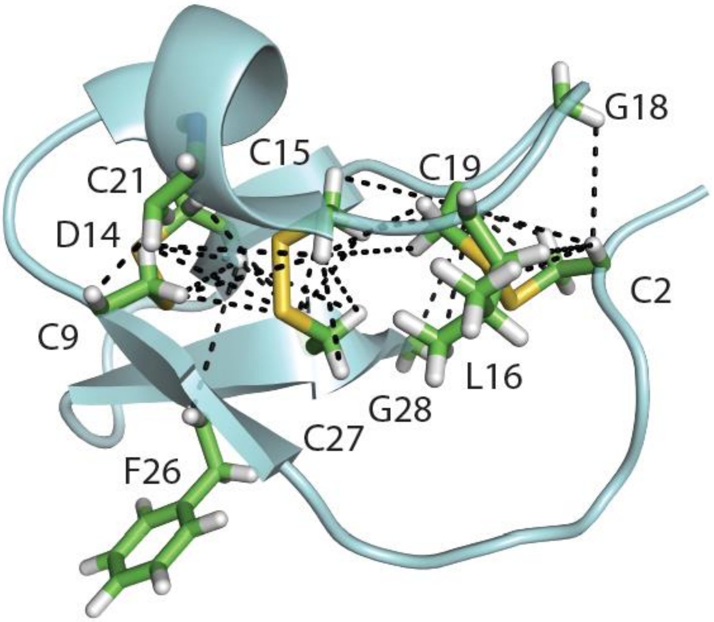


c

| Protons in the EETI structure 2IT7 | Qualification of the NOESY peak intensity | Distance in nm in 2IT7 |
|---|---|---|
| HA C27 - HB2/3 C15 | medium | 0.41/0.5 |
| HA C27 - HB3 C9 | medium | 0.47 |
| HA C27 - HB2 C21 | medium | 0.44 |
| | | |
| HA C21 - HA C27 | weak | 0.49 |
| HA C21 - HB3 C27 | overlap | 0.45 |
| HA C21 - HB2/3 C9 | overlap | 0.44/0.42 |
| HA C21 - HA F26 | weak | 0.49 |
| | | |
| HA C19 - HA2/3 G28 | weak | 0.55/0.46 |
| HA C19 - HA C2 | medium | 0.42 |
| HA C19 - HB2/3 C2 | strong | 0.23/0.37 |
| HA C19 - HB2/3 C15 | weak | 0.47/0.53 |
| | | |
| HA C15 - HB2/3 C27 | medium | 0.37/0.42 |
| HA C15 - HB2/3 C9 | weak | 0.43/0.53 |
| HA C15 - HB2/3 C19 | medium | 0.43/0.49 |
| | | |
| HA C9 - HB2/3 D14 | strong | 0.29/0.29 |
| | | |
| HA C2 - HA G18 | overlap | 0.37 |
| HA C2 - HB2/3 L16 | weak | 0.42/0.48 |
| HA C2 - HB2/3 C19 | strong | 0.23/0.37 |

**Supplementary Figure 2. NMR analysis of the cystine-knot fold in purified rEETI-II.** (a) The side chain fingerprint region of the experimental 300 ms mixing time 2D $^{1}H$-$^{1}H$ NOESY spectrum of rEETI-II (black, left) and the back-calculated NOESY based on the assignments of EETI-II structure (red, PDB entry 2IT7). The expected and observed cross peaks are marked in red in the experimental data set. Only signals relevant to the disulfide bonding patterns are considered. All expected NOESY peaks using a cutoff of 6 Å can be observed in the experimental data set, although some overlap makes the assignments ambiguous. (b) The distances between protons (calculated from the measured spectra) located in proximity to the C-S-S-C bonds are indicated by dotted lines and mapped onto the published structure of EETI-II (shown in ribbon diagram), with the cysteine residues represented as sticks. (c) Table of expected and observed distances and their intensities relevant to the disulfide bonding pattern.

a

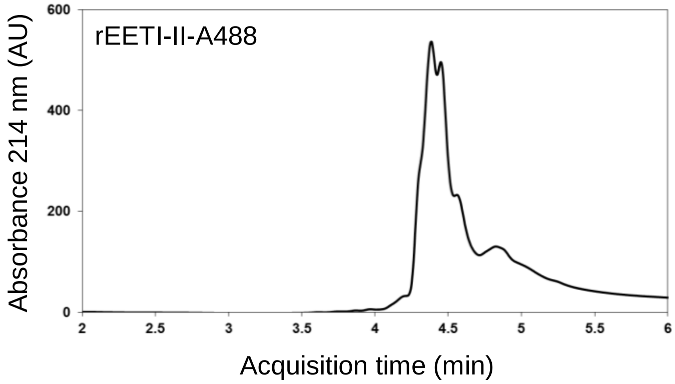


b

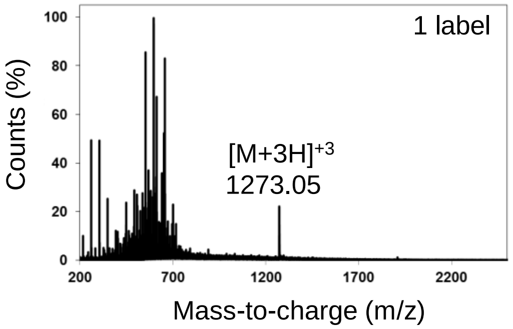


| Ion species | [M+3H]$^{+3}$ |
|---|---|
| **Expected MW** | **1272.26** |
| **Observed MW** | **1273.05** |

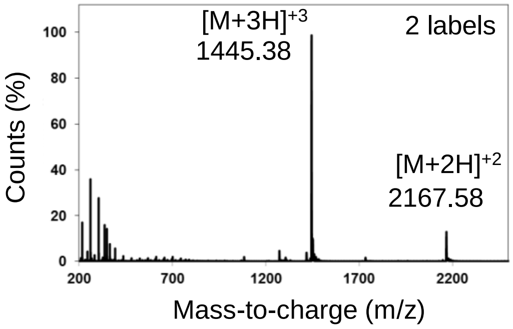


| Ion species | [M+2H]$^{+2}$ | [M+3H]$^{+3}$ |
|---|---|---|
| **Expected MW** | **2165.39** | **1443.93** |
| **Observed MW** | **2167.58** | **1445.38** |

**Supplementary Figure 3. LC-MS analysis of rEETI-II-A488.** Representative LC trace (a) and mass spectrometry analysis (b) of Alexa488-labeled rEETI-II. Alexa488 is a mixture of two isomers and EETI-II has two free amines for conjugation, explaining the multiple labeled peaks in the chromatogram.

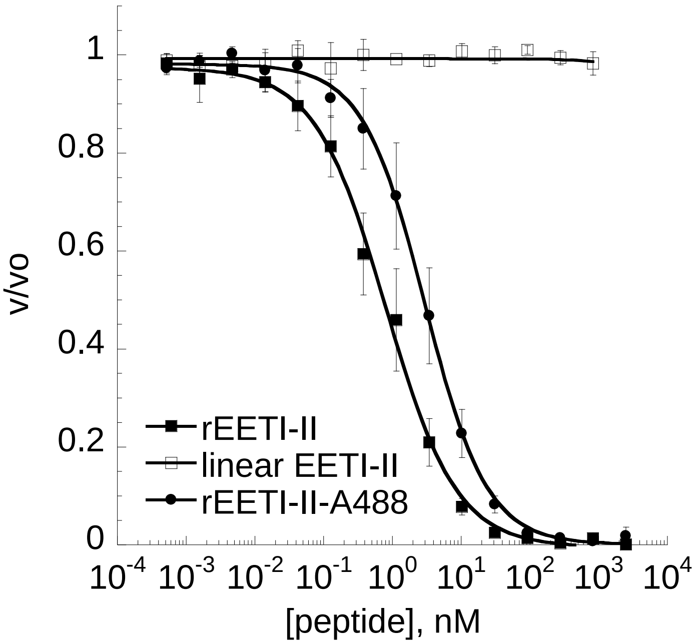


| | *K*i app (nM) |
|---|---|
| rEETI-II | 0.16 ± 0.01 |
| rEETI-II-A488 | 0.71 ± 0.06 |
| linear-EETI-II | no inhibition |

**Supplementary Figure 4. rEETI-II-A488 inhibits trypsin activity.**

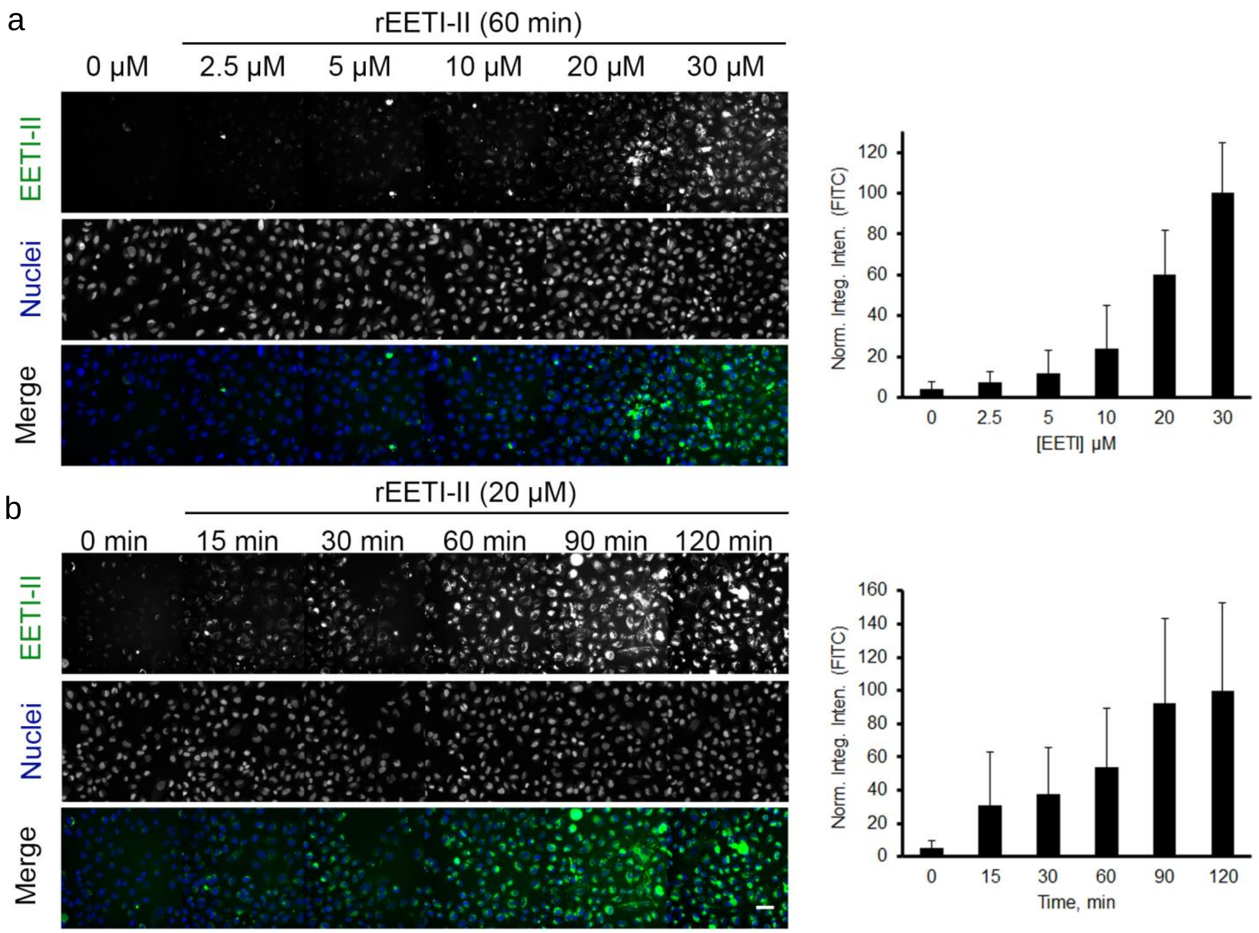


**Supplementary Figure 5. rEETI-II is internalized into mammalian cells. (a, b)** Concentration- and time-dependent uptake of fluorescently labeled rEETI-II. Incubation time and concentration are indicated in the figures. HeLa cells were incubated with rEETI-II-A488 then fixed with 4% PFA as described in methods. Fluorescence images were captured on a high throughput ImageXpress Micro XL imaging system (Molecular Devices) and images were analyzed by MetaXpress 4.0. Integrated fluorescence intensity values above a threshold defined using the DMSO-treated samples were measured and normalized to samples with the highest signal. Mean ± SD. n = 1,800 – 2,500 cells. Representative images from at least two independent experiments are shown. Scale bar, 50 µm.

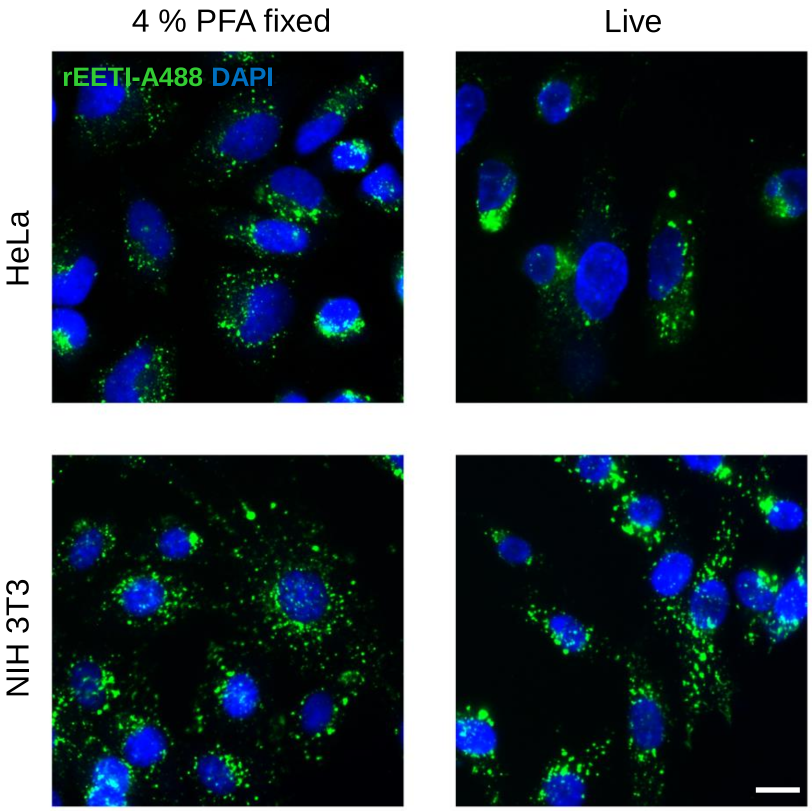


**Supplementary Figure 6. rEETI-A488 shows similar cellular localization in PFA fixed or live cells.** HeLa (upper panel) and NIH 3T3 (lower panel) cells were incubated with rEETI-II-A488 for 1 h then processed as described in methods. Fluorescence images were captured on a high throughput ImageXpress Micro XL imaging system (Molecular Devices). Representative images from at least two independent experiments are shown. Scale bar: 20 µm.

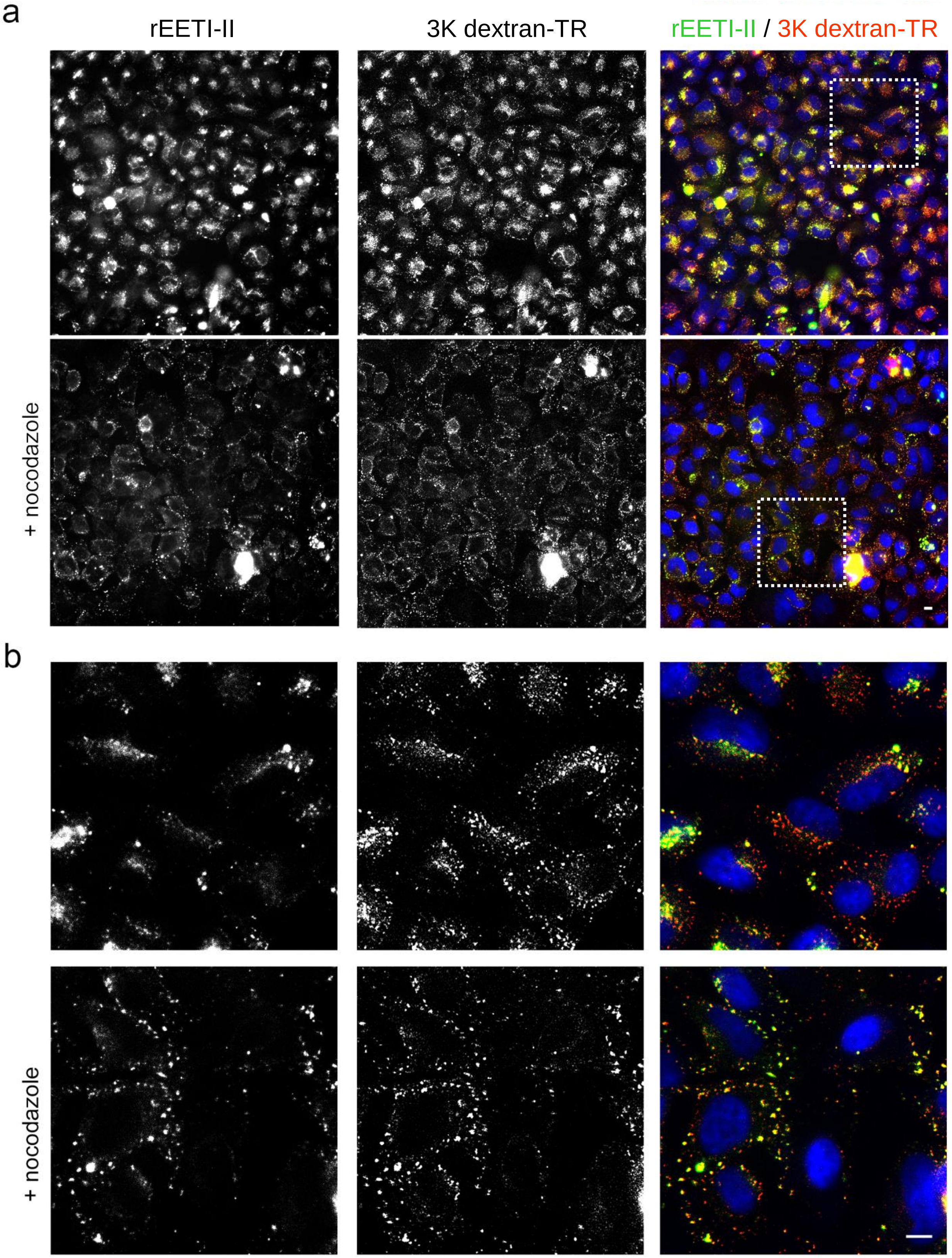


**Supplementary Figure 7. Cellular uptake of rEETI-II and 3K dextran.** (a-b) Representative images showing cellular distribution of rEETI-II-A488 (30 µM, 1 h) and 3K dextran conjugated to Texas red (0.1 mg/ml, 1 h) , in the presence or absence of 10 µm nocodazole (30 min pre-incubation followed by 60 min co-incubation with rEETI-II-A488). HeLa cells were fixed with 4% PFA and processed as described in methods. Fluorescence images were captured on a high throughput ImageXpress Micro XL imaging system (Molecular Devices). Representative images from at least two independent experiments are shown. Scale bar, 10 µm.

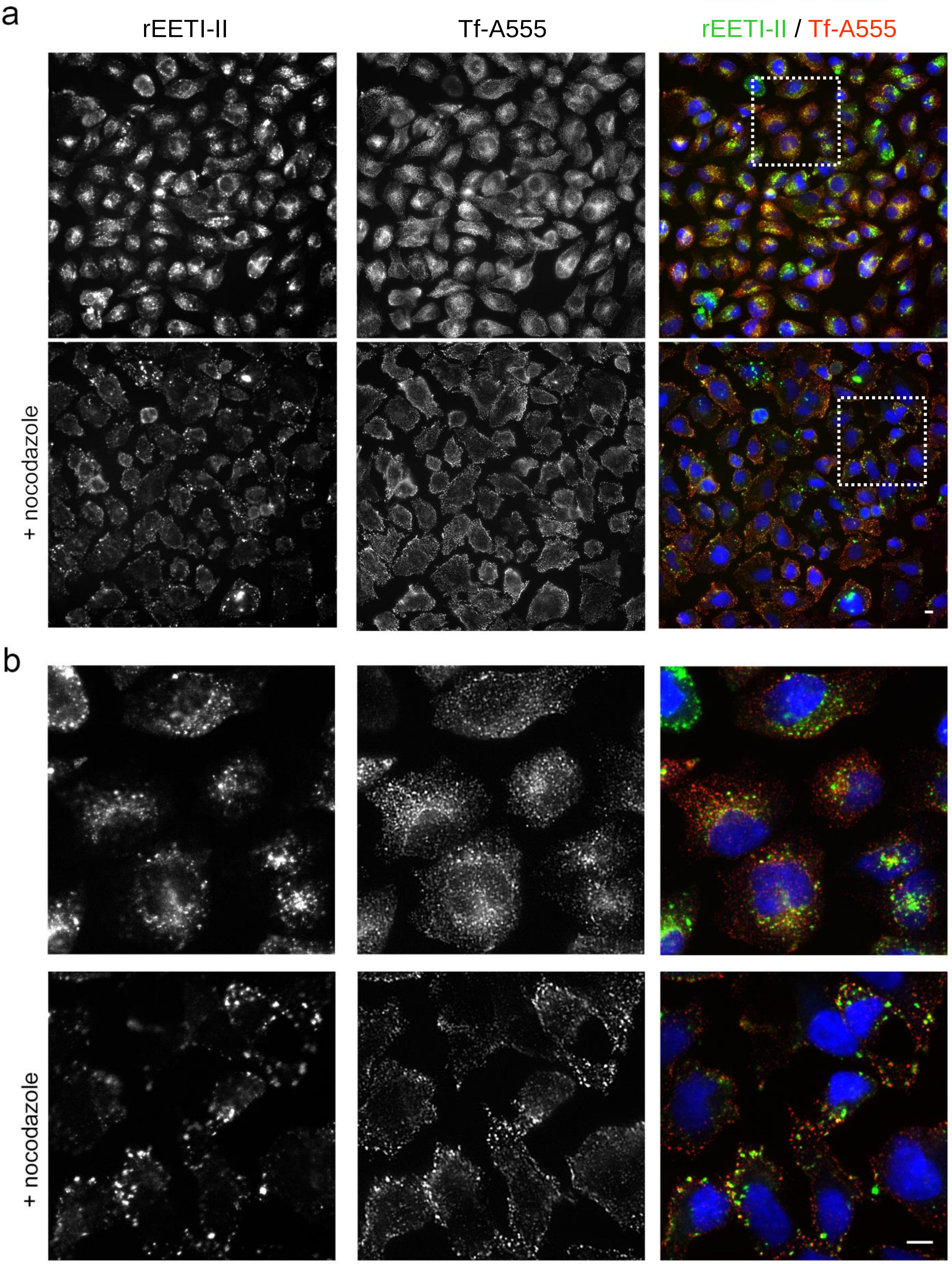


**Supplementary Figure 8. Cellular uptake of rEETI-II and Tf.** (a-b) Representative images showing cellular distribution of rEETI-II-A488 (30 µM, 1 h) and Tf-A555 (0.2 mg/ml, 10 min), in the presence or absence of 10 µm nocodazole (30 min pre-incubation followed by 60 min co-incubation with rEETI-II-A488). HeLa cells were fixed with 4% PFA and processed as described in methods. Fluorescence images were captured on a high throughput ImageXpress Micro XL imaging system (Molecular Devices). Representative images from at least two independent experiments are shown. Scale bar, 10 µm.

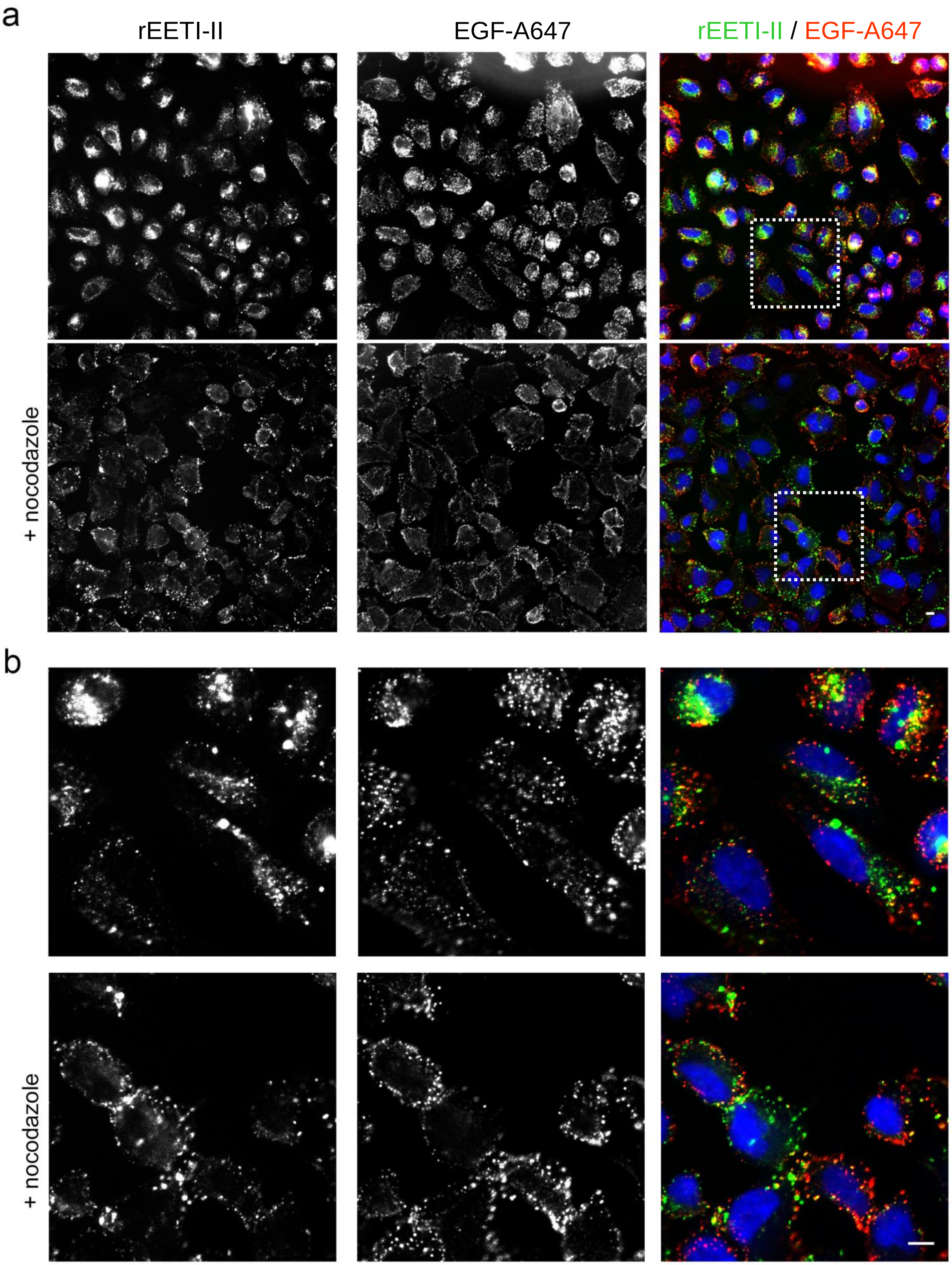


**Supplementary Figure 9. Cellular uptake of rEETI-II and EGF.** (a-b) Representative images showing cellular distribution of rEETI-II-A488 (30 µM, 1 h) and EGF-A647 (5 µg/ml, 10 min), in the presence or absence of 10 µm nocodazole (30 min pre-incubation followed by 60 min co-incubation with rEETI-II-A488). HeLa cells were fixed with 4% PFA and processed as described in methods. Fluorescence images were captured on a high throughput ImageXpress Micro XL imaging system (Molecular Devices). Representative images from at least two independent experiments are shown. Scale bar, 10 µm.

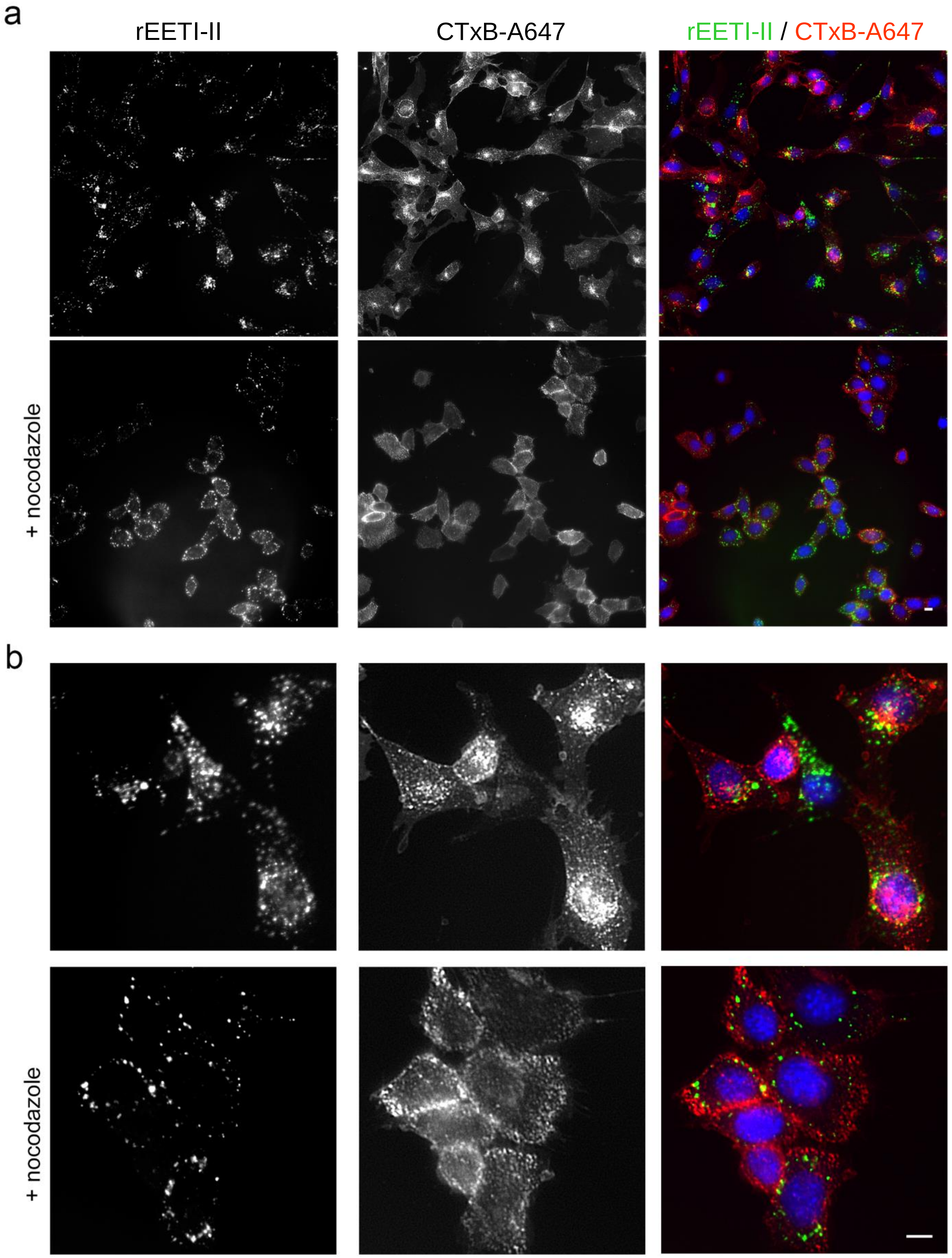


**Supplementary Figure 10. (a-b) Cellular uptake of rEETI-II and cholera toxin subunit b (CTxB).** Representative images showing cellular distribution of rEETI-II-A488 (30 µM, 1 h) and CTxB-A647 conjugated (25 µg/ml, 10 min), in the presence or absence of 10 µm nocodazole (30 min pre-incubation followed by 60 min co-incubation with rEETI-II-A488). NIH 3T3 cells were fixed with 4% PFA and processed as described in methods. Fluorescence images were captured on a high throughput ImageXpress Micro XL imaging system (Molecular Devices). Representative images from at least two independent experiments are shown. Scale bar, 10 µm.

c

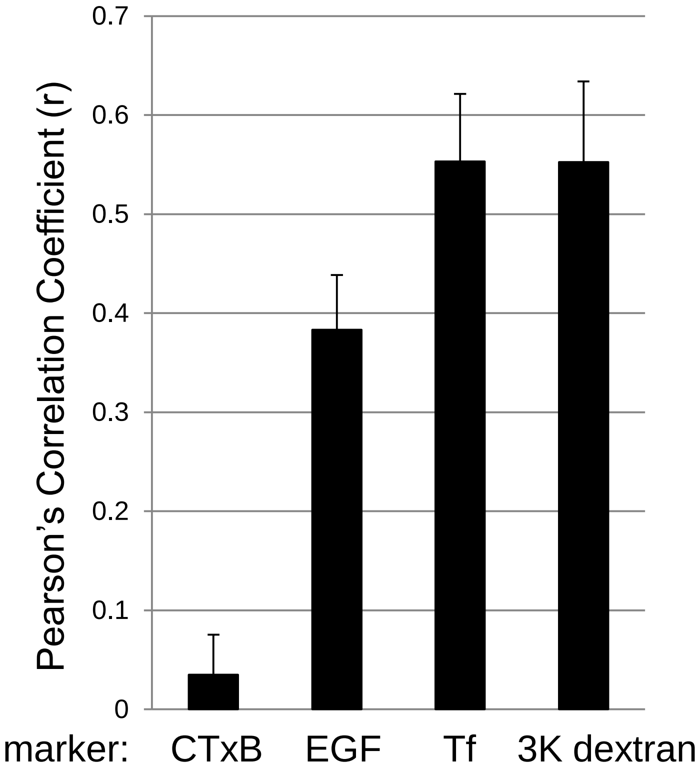


**Supplementary Figure 10. (c) rEETI-II-A488 co-localizes with internalized Tf, EGF, and 3K dextran-positive puncta, but not CTxB.** Colocalization of rEETI-II-A488 and markers wes reported using Pearson's correlation coefficient (r) win ImageJ (Fiji). Mean ± SD. n = 60 - 80 cells.

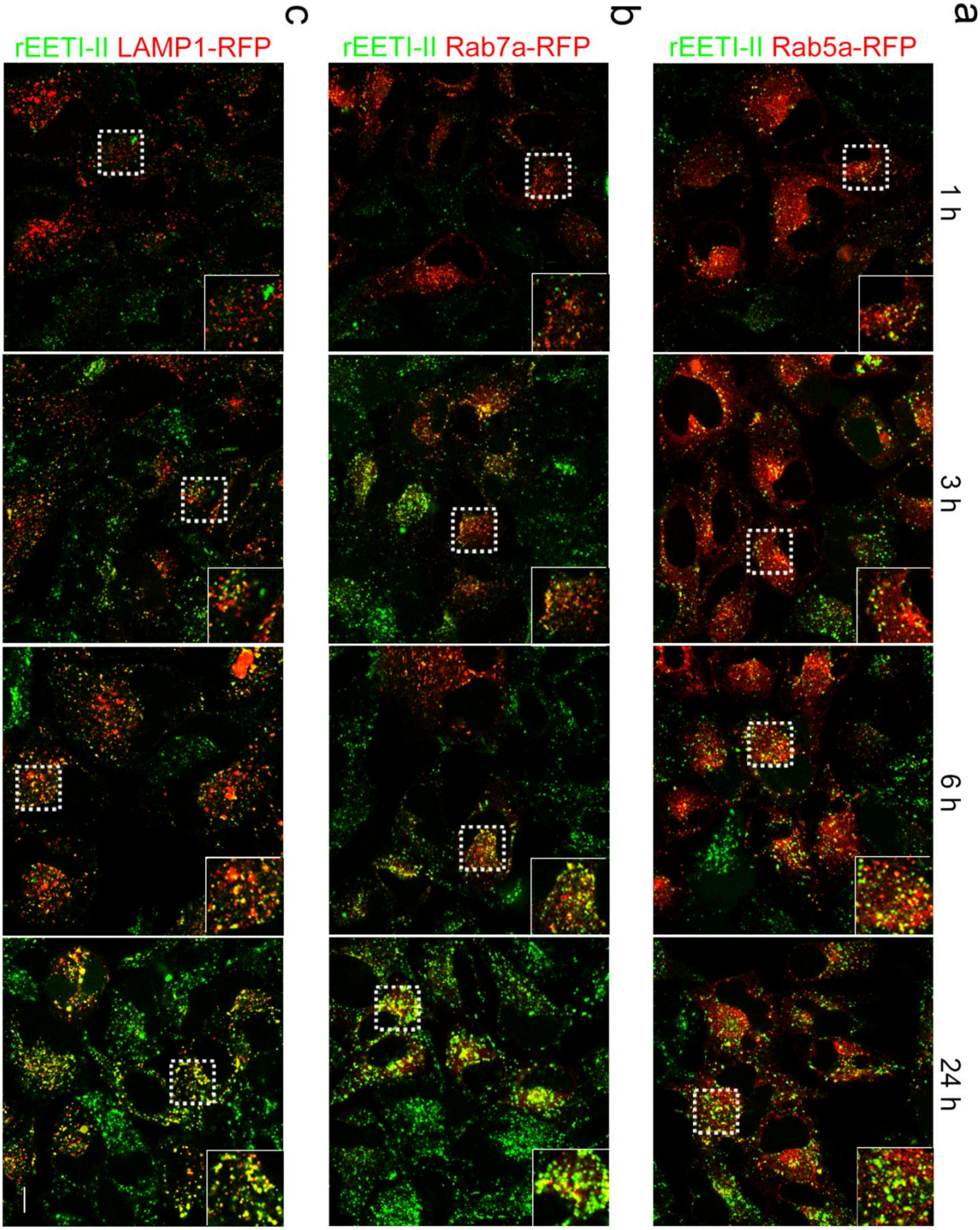


**Supplementary Figure 11. Internalized rEETI-II co-localizes with Rab5a, Rab7 or LAMP1 in HeLa cells in a time-dependent manner.** (a-c) rEETI-II co-localizes with Rab5a (early endosome marker), Rab7 (late endosome marker) or LAMP1 (lysosome marker). HeLa cells over expressing Rab5a-RFP, Rab7a-RFP or LAMP1-RFP (all shown in red) were incubated with rEETI-II-A488 (10 µM, shown in green) at 37 °C. Cells were washed at various time points and fixed with 4% PFA as described in methods. Cell images were captured on an inverted LEICA SP5 laser scanning confocal microscope (Leica Microsystems). Representative images from at least two independent experiments are shown. Scale bar, 10 µm.

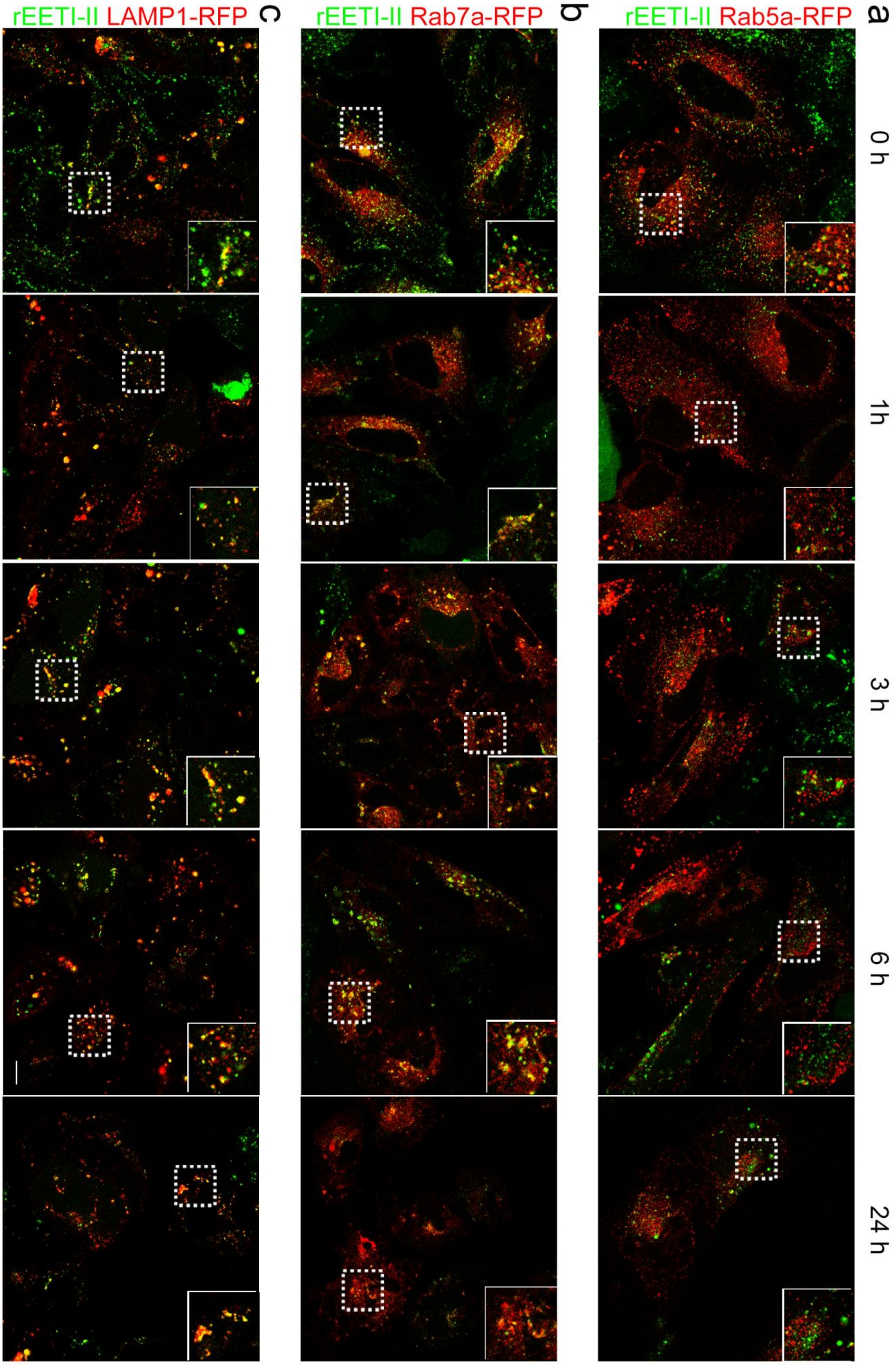


**Supplementary Figure 12. Pulse chase experiment of rEETI-II.** See legend on next page.

**Supplementary Figure 12. Pulse chase experiment of rEETI-II.** (a-c) rEETI-II co-localizes with Rab5a (early endosome marker), Rab7 (late endosome marker) or LAMP1 (lysosome marker). HeLa cells over expressing Rab5a-RFP, Rab7a-RFP or LAMP1-RFP (all shown in red) were incubated with rEETI-II-A488 (10 µM, shown in green) for 3 h then washed and incubated with complete medium at 37 °C. Cells were then washed at various time points and fixed with 4% PFA as described in methods. Images were captured on an inverted LEICA SP5 laser scanning confocal microscope (Leica Microsystems). Representative images from at least two independent experiments are shown. Scale bar, 10 µm.

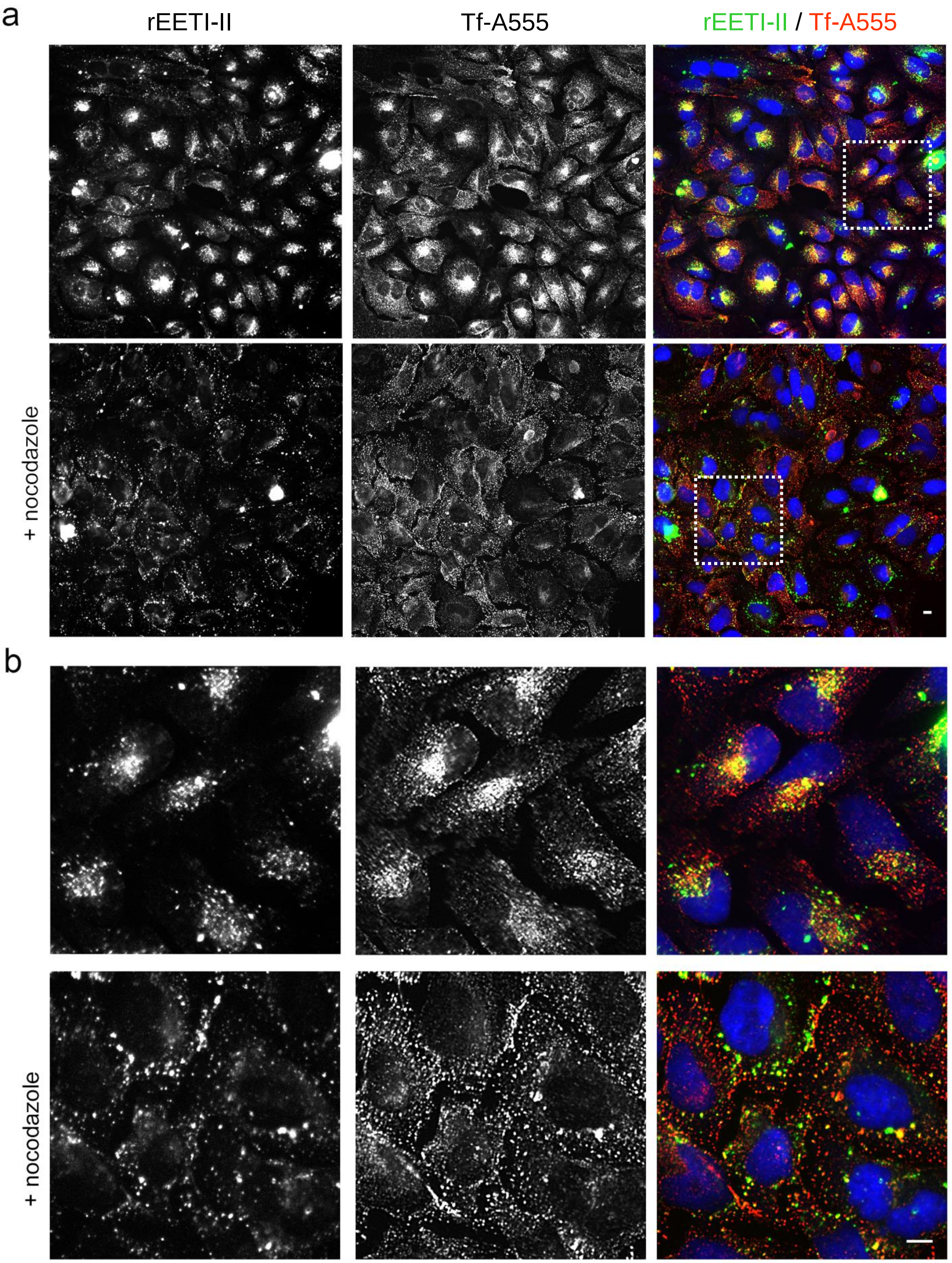


**Supplementary Figure 13 . Colocalization of internalized rEETI-II and Tf at recycling endosomes.** (a-b) Representative images showing cellular distribution of rEETI-II-A488 (30 µM, 3 h) and Tf-A555 (0.2 mg/ml, 3 h), in the presence or absence of 10 µm nocodazole (30 min pre-incubation followed by 60 min co-incubation with rEETI-II-A488). HeLa cells were fixed with 4% PFA and processed as described in methods. Fluorescence images were captured on a high throughput ImageXpress Micro XL imaging system (Molecular Devices). Representative images from at least two independent experiments are shown. Scale bar, 10 µm.

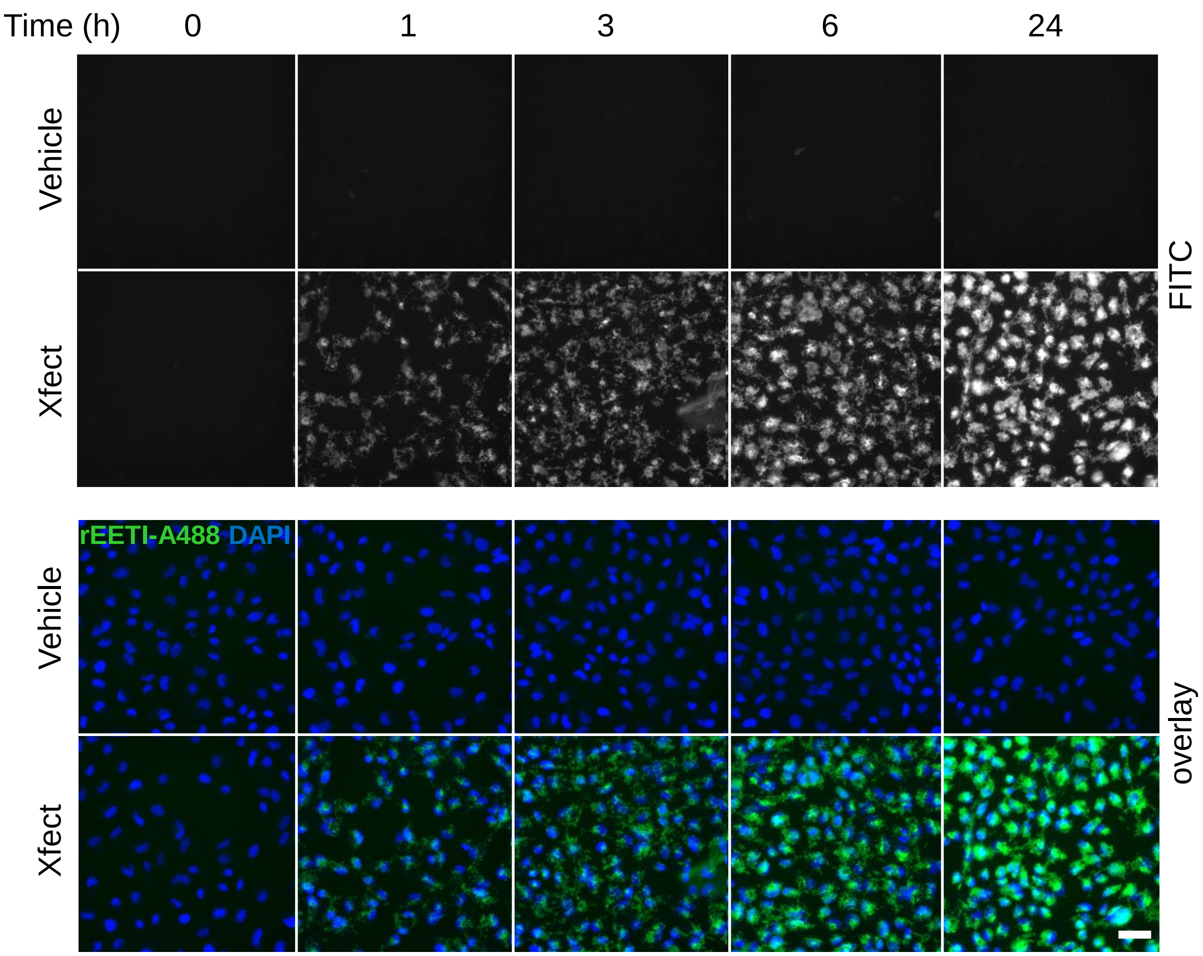


**Supplementary Figure 14. A cell-penetrating peptide enhances the cellular uptake of rEETI-II.** HeLa cells were treated with the rEETI-II-A488/Xfect complexes or rEETI-II-A488 alone (1 µM) for the indicated times then washed and fixed with 4% PFA as described in methods. Fluorescence images were captured on a high throughput ImageXpress Micro XL imaging system (Molecular Devices). Representative images from at least two independent experiments are shown. Scale bar, 50 µm.

a

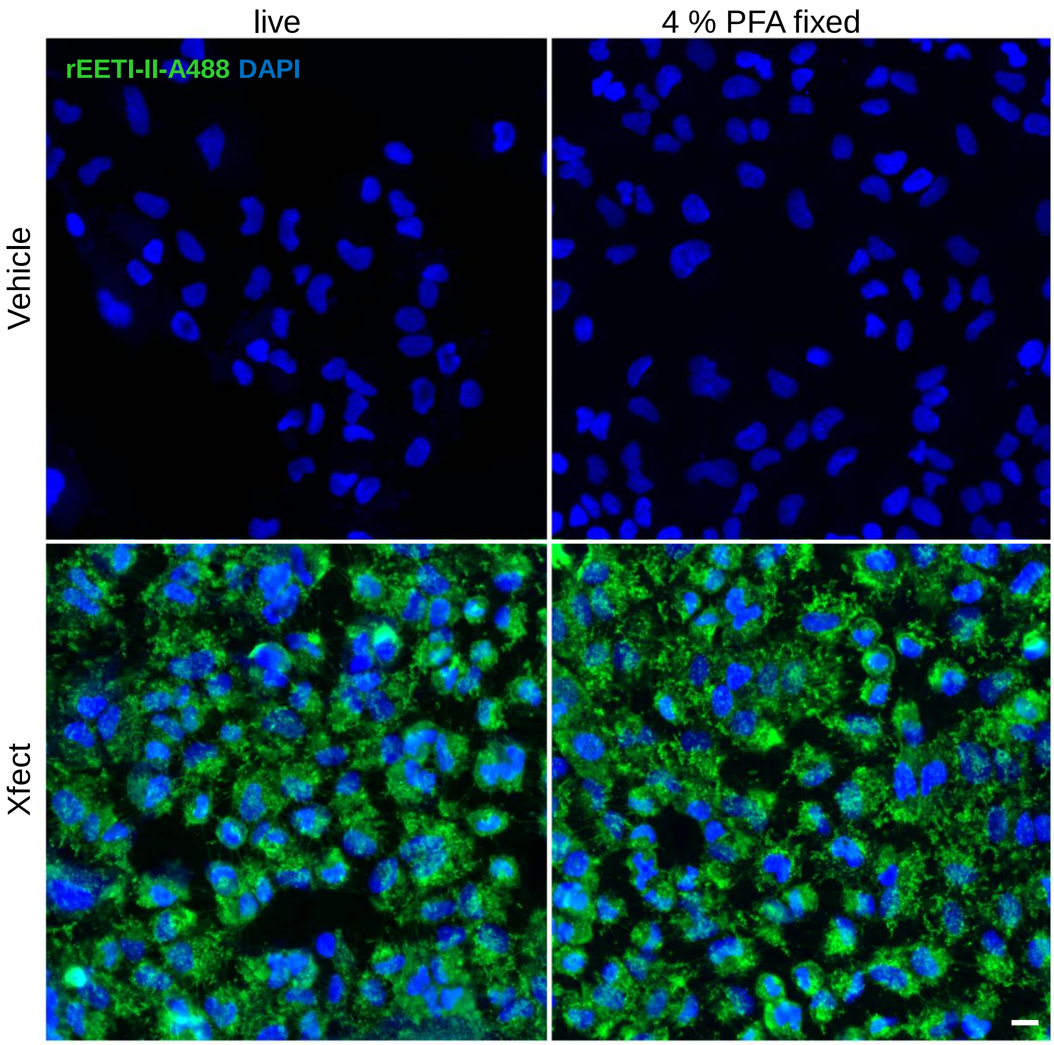


**Supplementary Figure 15. A cell-penetrating peptide enhances the cellular uptake of rEETI-II and alters its distribution pattern.** (a) Co-incubation of rEETI-II-A488 (5 µM) with a cell-penetrating peptide (Xfect) enhances uptake efficiency of rEETI-II in live or fixed cells. HeLa cells were treated with rEETI-II-A488 (5 µM) in the absence or presence of Xfect for 6 h then processed as described in methods. Fluorescence images were captured on a high throughput ImageXpress Micro XL imaging system (Molecular Devices). (b-c) The cell-penetrating peptide Xfect alters the cellular distribution of rEETI-II. Images of cell treated with rEETI-II-A488 alone (panel c) were adjusted for brightness to show its cellular distribution. Representative images from at least two independent experiments are shown. Scale bar: 20 µm.

b

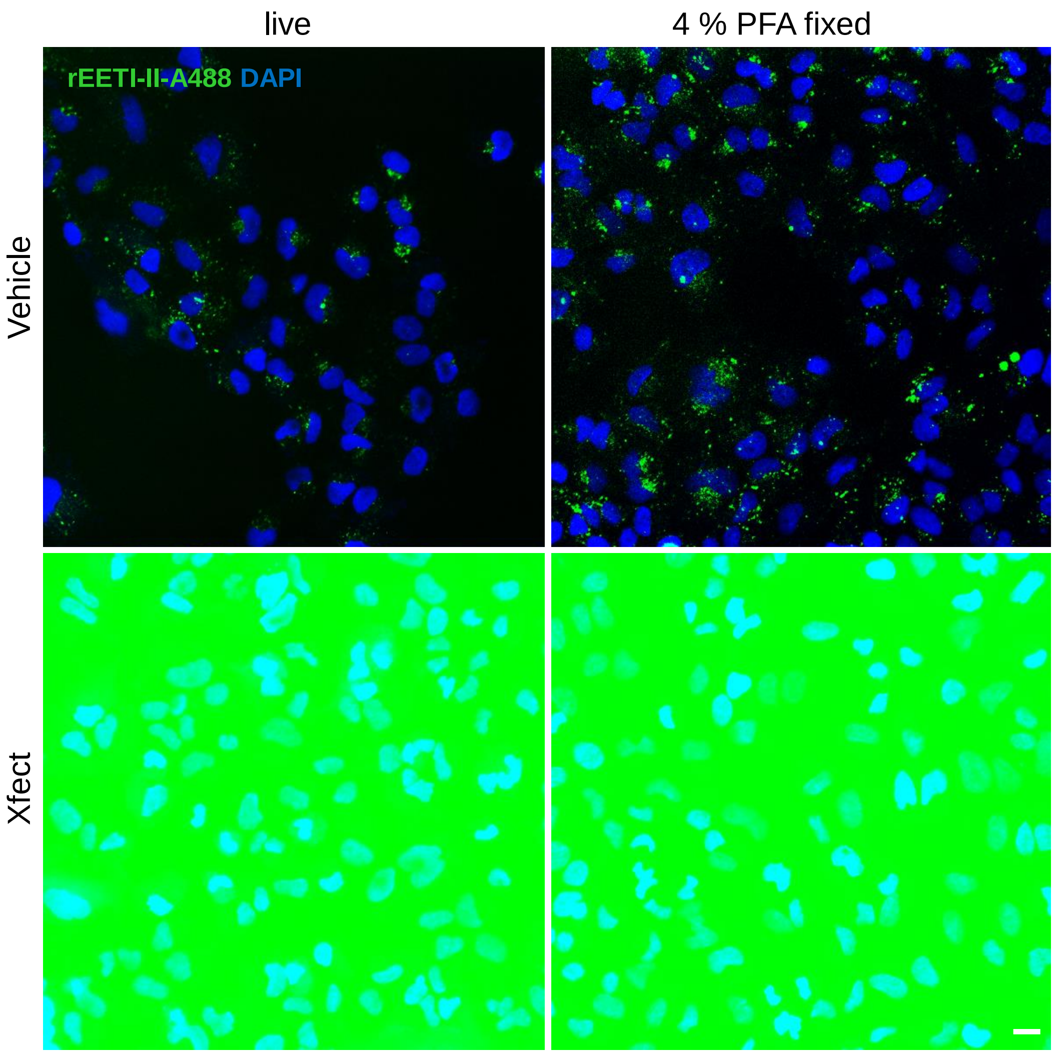

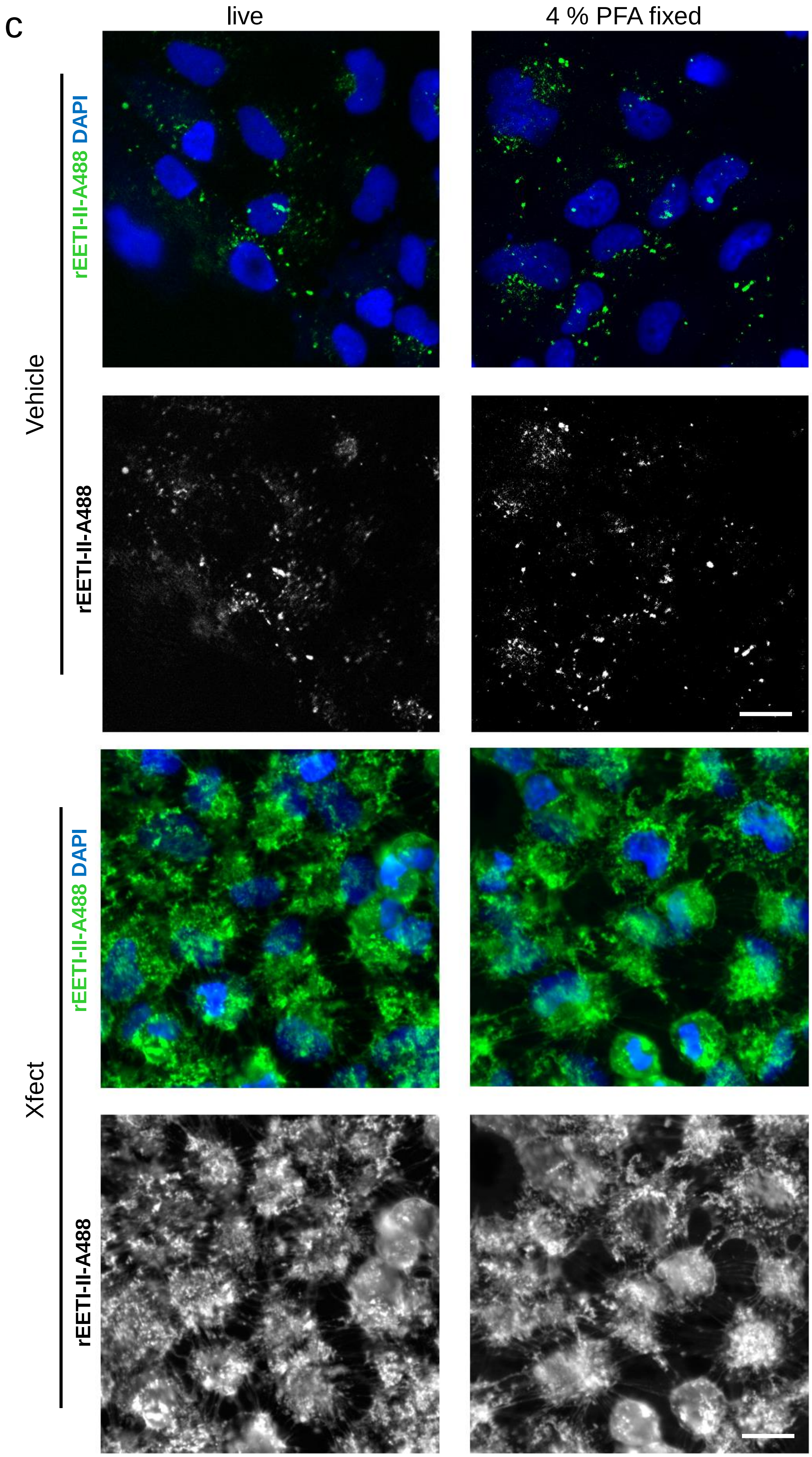
c
live
4 % PFA fixed
Vehicle
rEETI-II-A488 DAPI
rEETI-II-A488
Xfect
rEETI-II-A488 DAPI
rEETI-II-A488

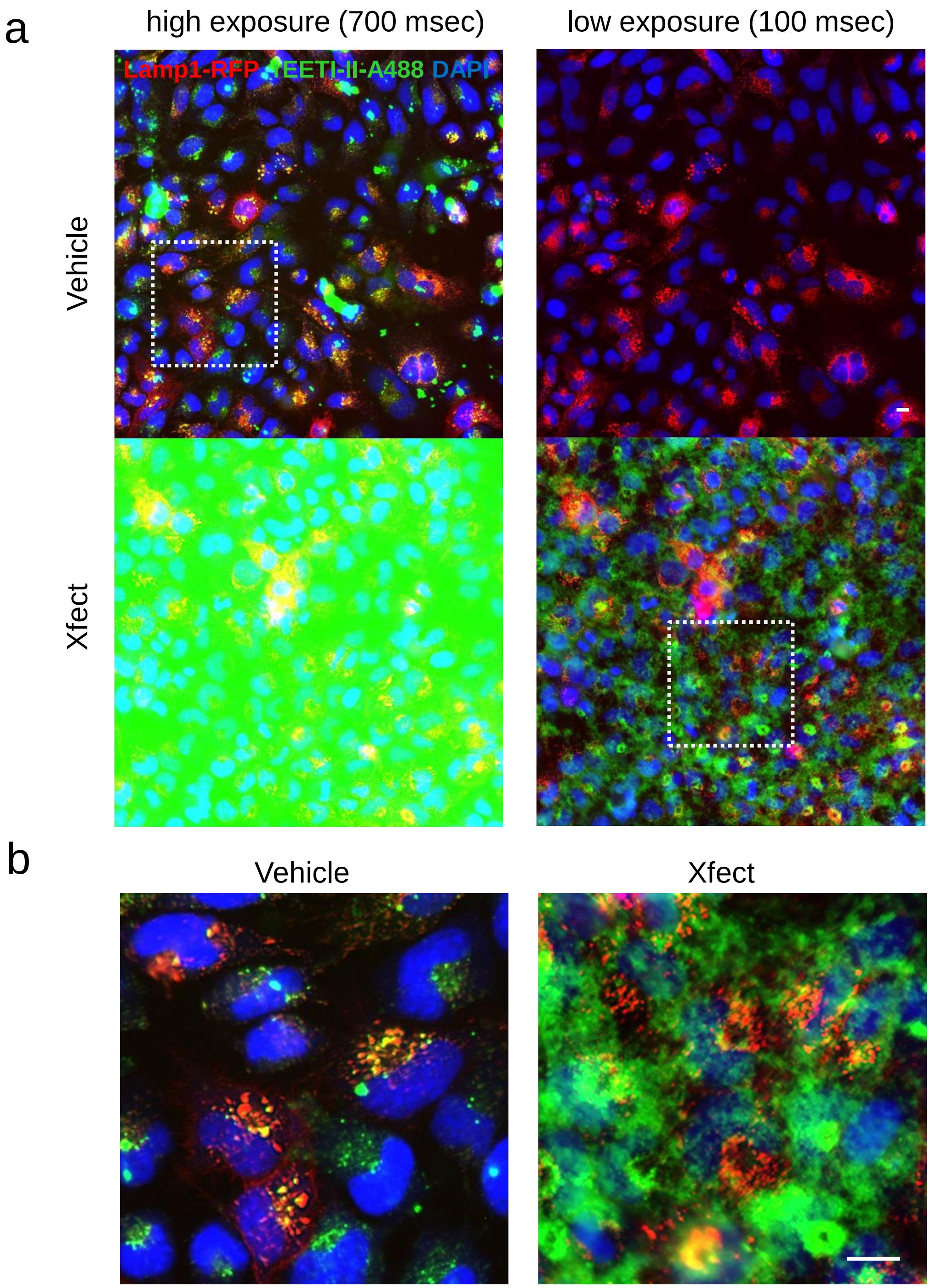


**Supplementary Figure 16. A cell-penetrating peptide alters subcellular distribution of rEETI-II.** HeLa cells were infected with Lamp1-RFP for 24 h then treated with rEETI-II-A488 (5 µM) in the absence or presence of Xfect for 6 h. Cells were then fixed with 4% PFA as described in methods. Images of cells treated with rEETI-II-A488 alone were overexposed (700 ms vs 100 ms) to show its cellular distribution. Fluorescence images were captured on a high throughput ImageXpress Micro XL imaging system (Molecular Devices). Images were analyzed by MetaXpress 4.0. Percentage of FITC (rEETI-II-A488) area colocalized with LAMP1 area (both intensity set above a threshold defined using the DMSO-treated samples) were measured and normalized to the percentage of LAMP1-positive cells. Mean ± SD. n = 1,600 cells. Representative images from at least four independent experiments are shown. Scale bar, 20 µm.

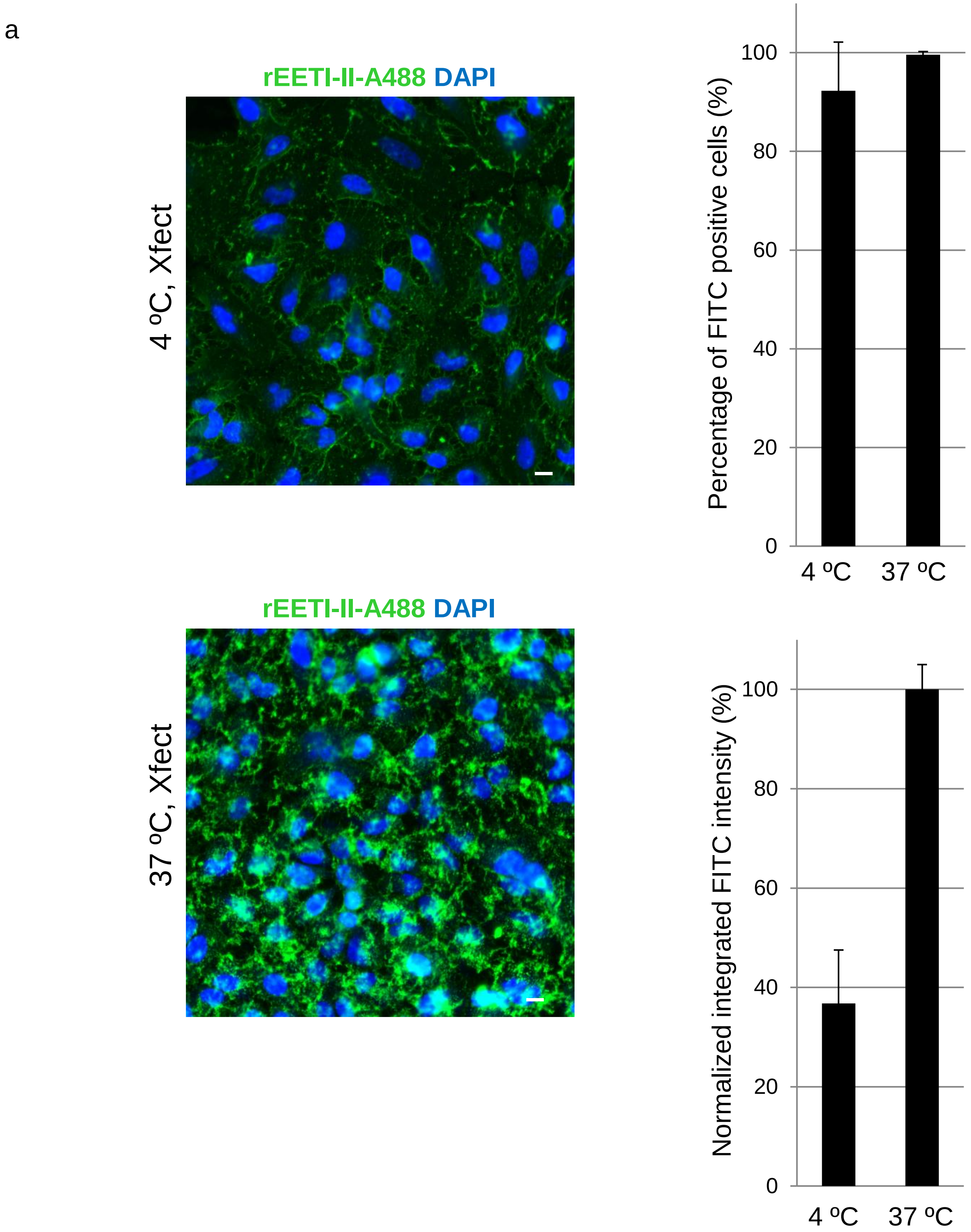


**Supplementary Figure 17. Uptake of rEETI-II/Xfect is temperature-dependent.** HeLa cells were treated with rEETI-II-A488 (5 µM) preincubated with (a) or without (b) Xfect for 1 hour at 37 ° C or 4 ° C. Cells were washed with PBS at the end of the study and fixed with 4% PFA. Fluorescence images were captured on a high throughput ImageXpress Micro XL imaging system (Molecular Devices). Images were analyzed by MetaXpress 4.0. Mean ± SD. n = 500 cells. Representative images from two independent experiments are shown. Exposure time: 400 msec (panel a) and 900 msec (panel b). Panel b images are shown both in color and back and white. Scale bar, 20 µm.

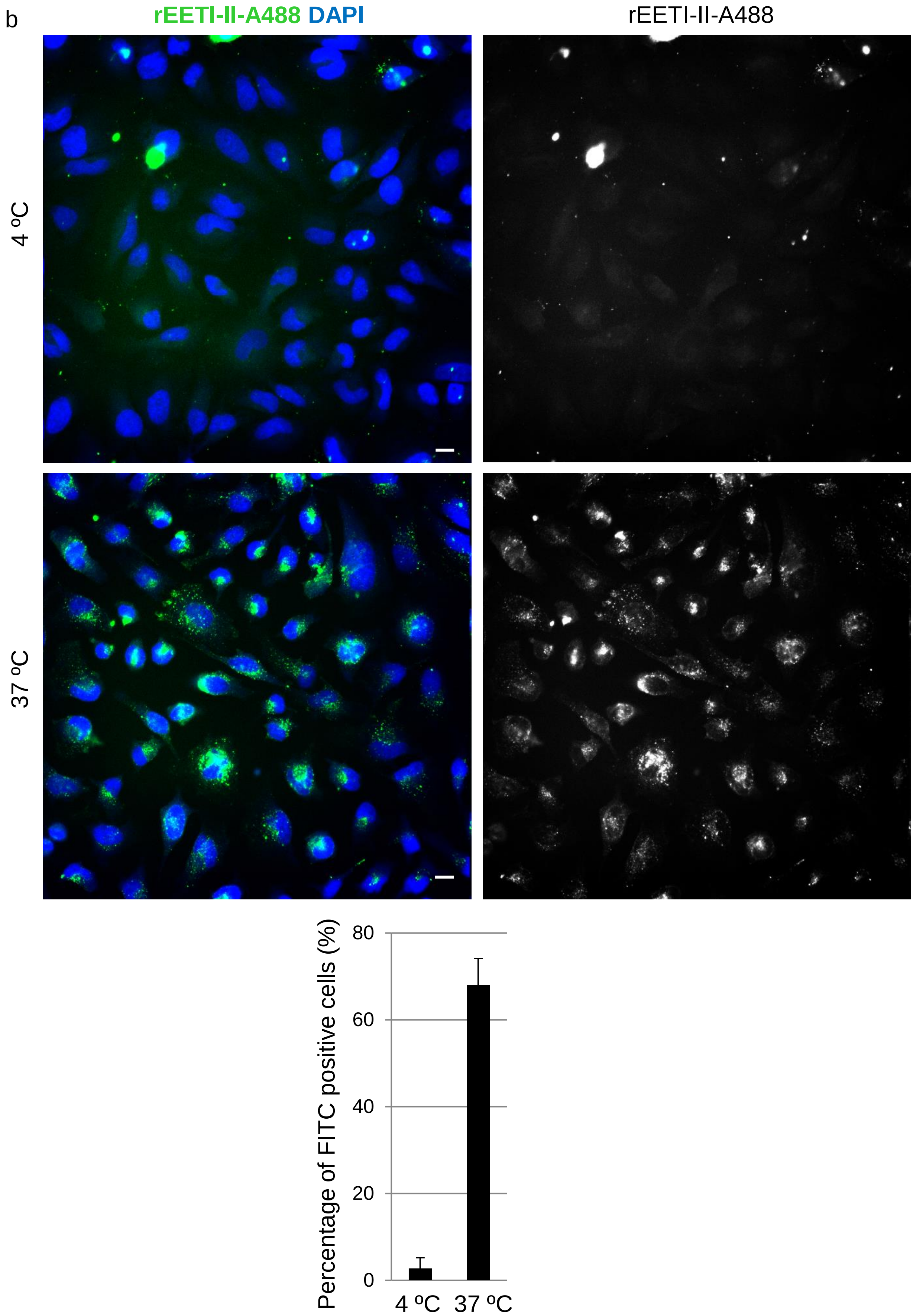


**Supplementary Figure 17. Uptake of rEETI-II/Xfect is temperature-dependent.** Continued.

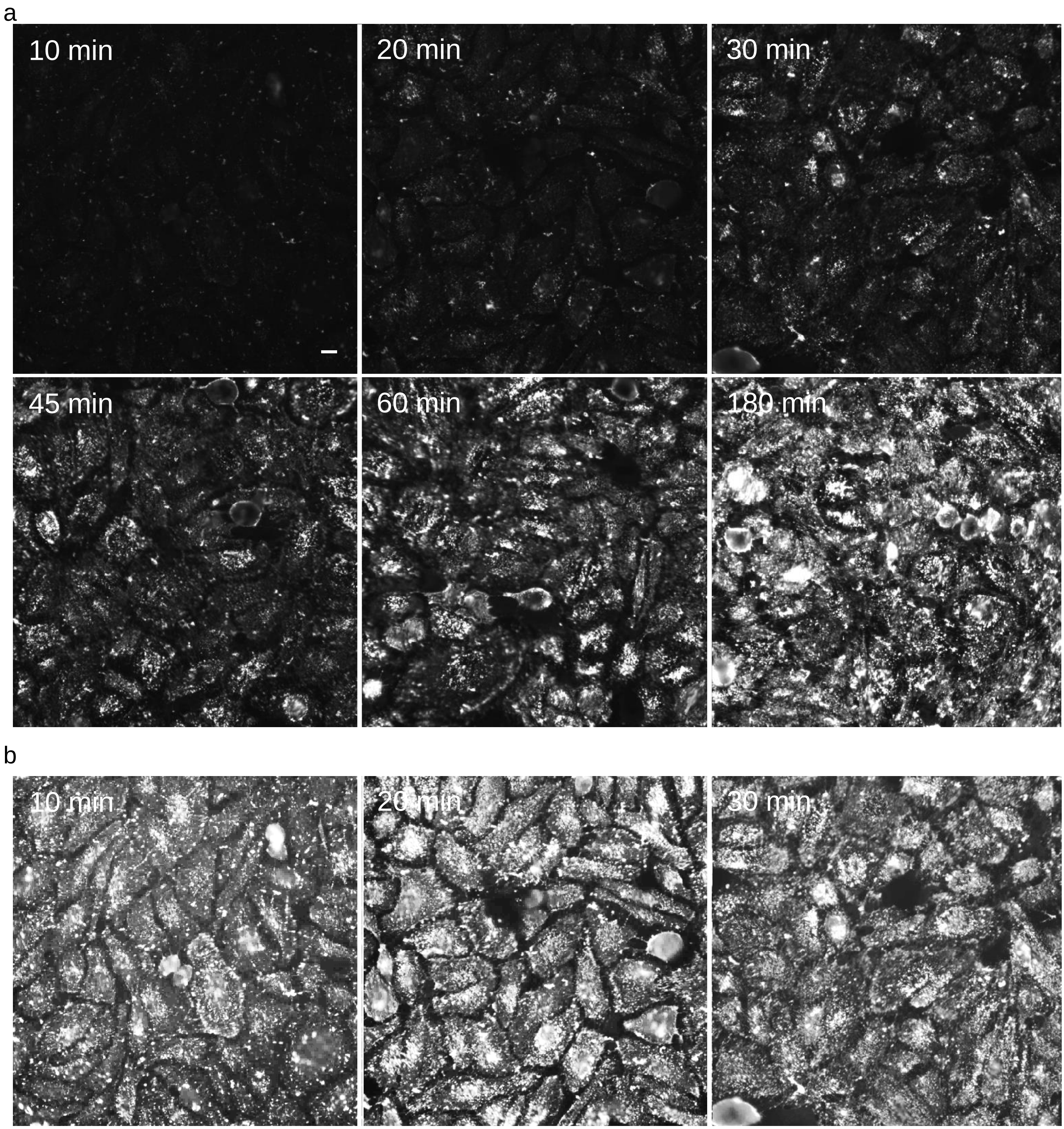


**Supplementary Figure 18. Uptake and subcellular distribution of rEETI-II co-incubated with Xfect is time-dependent.** HeLa cells were treated with rEETI-II-A488 (1 µM) preincubated with Xfect. Cells were washed at various time points and fixed with 4% PFA as described in methods. Fluorescence images were captured on a high throughput ImageXpress Micro XL imaging system (Molecular Devices). Representative images from at least three independent experiments are shown. Scale bar, 20 µm. (b) Images from the top panel of (a) are normalized differently to show the subcellular distribution of rEETI-II/Xfect at early time points.

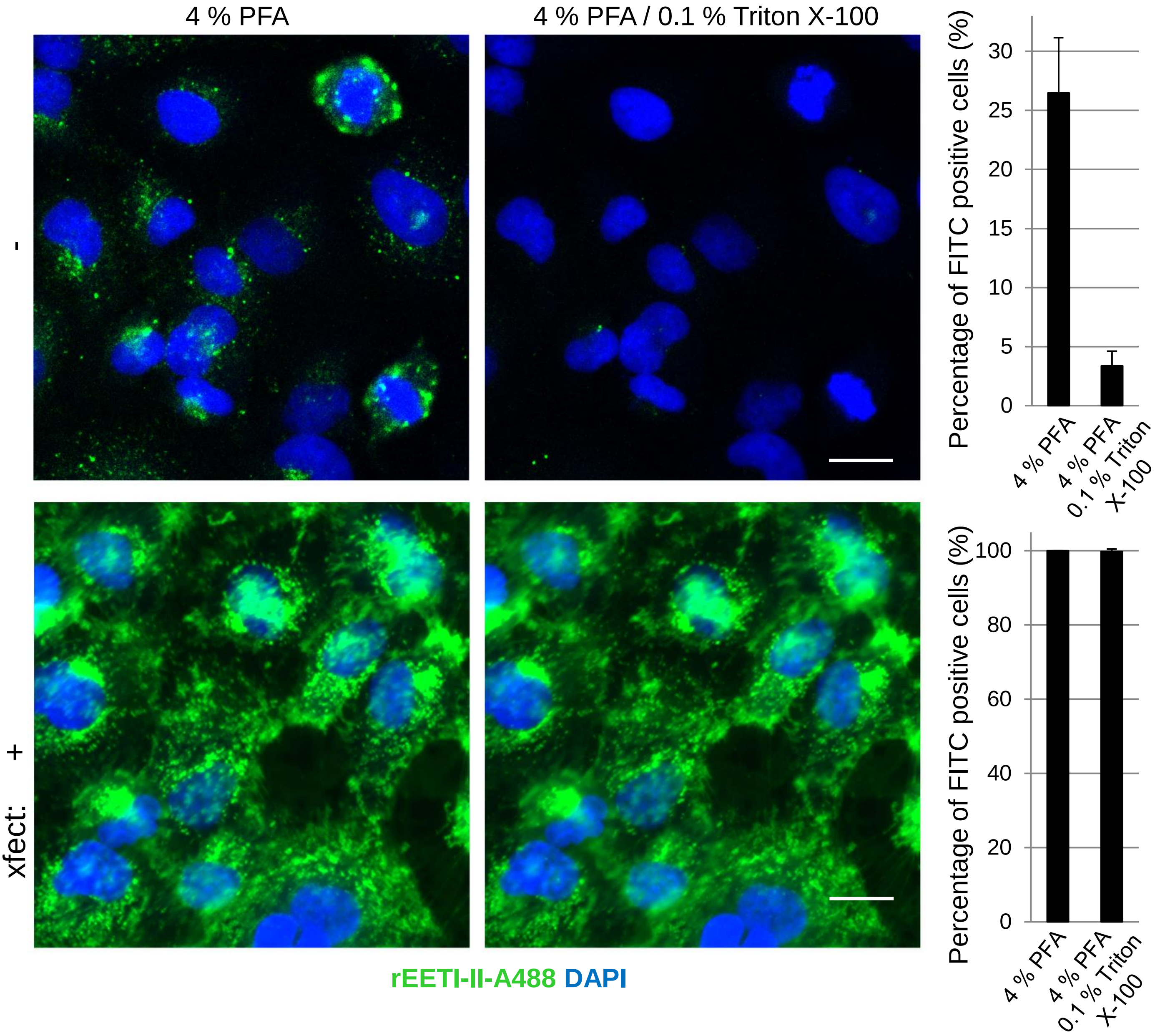


**Supplementary Figure 19. The rEETI-II/Xfect complex, but not rEETI-II alone, maintains its subcellular staining upon detergent permeabilization.** HeLa cells were treated with rEETI-II-A488 (5 µM) in the absence or presence of Xfect for 3 h then fixed with 4% PFA as described in methods. After imaging, cells were permeabilized with 0.1 % Triton X-100 in PBS for 5 min at room temperature, washed and imaged again. Images of cell treated with rEETI-II-A488 alone were overexposed (500 ms vs 100 ms) to show its cellular distribution. Fluorescence images were captured on a high throughput ImageXpress Micro XL imaging system (Molecular Devices). Mean ± SD. n = 900 cells. Representative images from at least two independent experiments are shown. Scale bar, 20 µm.
